# Chemical and physical restraint use during acute care hospitalization of older adults: A retrospective cohort study and time series analysis

**DOI:** 10.1371/journal.pone.0276504

**Published:** 2022-10-26

**Authors:** Aaron Jones, Zahra Goodarzi, Justin Lee, Richard Norman, Eric Wong, Monidipa Dasgupta, Barbara Liu, Jennifer Watt

**Affiliations:** 1 Department of Health Research Methods, Evidence, and Impact, McMaster University Hamilton, Hamilton, Ontario, Canada; 2 ICES, Toronto, Ontario, Canada; 3 Division of Geriatric Medicine, Department of Medicine, University of Calgary, Calgary, Alberta, Canada; 4 Department of Community Health Sciences, University of Calgary, Calgary, Alberta, Canada; 5 Hotchkiss Brain Institute, University of Calgary, Calgary, Alberta, Canada; 6 O’Brien Institute of Public Health, University of Calgary, Calgary, Alberta, Canada; 7 Division of Geriatric Medicine, Department of Medicine, McMaster University, Hamilton, Ontario, Canada; 8 Division of Geriatric Medicine, Department of Medicine, University of Toronto, Toronto, Ontario, Canada; 9 Knowledge Translation Program, Li Ka Shing Knowledge Institute, St Michael’s Hospital, Toronto, Ontario, Canada; 10 Division of Geriatric Medicine, Department of Medicine, Western University, London, Ontario, Canada; Icahn School of Medicine at Mount Sinai, UNITED STATES

## Abstract

**Background:**

Chemical and physical restraints are associated with harm in older adults, but our understanding of their use during acute care hospitalizations is limited.

**Objectives:**

To (1) describe restraint use during acute care hospitalizations of older adults at the onset of the COVID-19 pandemic compared to pre-pandemic levels and (2) describe between-hospital variability in restraint use.

**Design:**

Retrospective cohort study with a time series analysis.

**Participants:**

Acute care hospital inpatients, aged 65 years or older, who were discharged from one of four Alberta hospitals or six Ontario hospitals in Canada, between November 1, 2019, and June 30, 2020.

**Main measures:**

We used autoregressive linear models with restricted cubic splines to compare proportions of chemical restraint (that is, psychotropic medications, namely antipsychotics, benzodiazepines, and trazodone) and physical restraint (e.g., mittens) use immediately after the onset of the COVID-19 pandemic with pre-pandemic levels. We describe between-hospital variability in restraint use using intraclass correlation coefficients (ICC) and median odds ratios (OR).

**Key results:**

We included 71,004 hospitalizations. Adjusted for the prevalence of dementia and psychotic disorders, chemical restraint use increased in Ontario hospitals from a pre-pandemic average of 27.1% to 30.8% (p<0.001) before returning to pre-pandemic levels within eight weeks. Physical restraint orders in Ontario increased from 5.9% to 8.3% (p = 0.012) and remained elevated at eight weeks. No significant changes in restraint use were observed in Alberta. There was moderate between-hospital variability in chemical restraint use (ICC 0.041 and median OR 1.43). Variability in physical restraint use was higher (ICC 0.11 and median OR 1.83).

**Conclusions:**

The COVID-19 pandemic impacted in-hospital use of chemical and physical restraints among older adults in Ontario but not Alberta. Substantial differences in chemical and physical restraint use by region and hospital suggests there are opportunities to improve best practices in geriatric care. Future research must support implementation of evidence-informed interventions that standardize appropriate restraint use.

## Introduction

Seventeen to 20% of adults aged 65 years or older experience one or more acute care hospitalizations each year [1, 2]. Up to 27% of older adults admitted to a medical unit are physically restrained (e.g., with a lap belt or mittens) and 32% are prescribed a psychotropic medication (that is, chemical restraint) [3, 4]. Chemical and physical restraints should only be used as a last resort because they are associated with potential harms in older adults [5, 6]. Chemical restraints are associated with an increased risk of death, strokes, falls, and fractures; and physical restraints are associated with functional decline and death [7–11]. Despite the risks associated with using chemical and physical restraints, restraint appropriateness, consent for using restraints, and use of nonpharmacologic interventions prior to implementing restraints, are inconsistently documented in medical records [5, 6, 12].

There is substantial literature documenting chemical restraint use and describing interventions to improve their appropriateness in nursing homes [13–16]. Chemical restraint use increased in nursing homes during the COVID-19 pandemic [13]. However, less is known about how chemical and physical restraints are used during acute care hospitalizations of older adults and whether these restraints are appropriate [3, 17–19]. This is an important knowledge gap given the potential for chemical and physical restraints to cause harm [8, 10]. Further, having a better understanding of chemical and physical restraint use during acute care hospitalizations of older adults, both before and after the onset of COVID-19 pandemic related restrictions, will facilitate the development of interventions that improve their appropriate use. Our objectives were to (1) describe changes in chemical and physical restraint use among older adults admitted to acute care hospitals in Alberta and Ontario, Canada, immediately after the onset of public health restrictions related to the COVID-19 pandemic and (2) describe between-hospital variability in chemical and physical restraint use.

## Methods

We reported our findings as per STROBE (Strengthening the reporting of observational studies in epidemiology) and RECORD (Reporting of studies conducted using observational routinely collected data) statements [20, 21].

### Study design, setting, data sources, and population

We conducted a time series analysis of acute care inpatients, aged 65 years or older, who were discharged from one of six Ontario hospitals or four Alberta hospitals (in Canada) between November 1, 2019, and June 30, 2020. Canada has a publicly funded health care system with universal health insurance for residents that includes physician services and hospitalization-related costs. We excluded individuals admitted to inpatient psychiatry. Hospitals were in Ontario (cities of Toronto, Hamilton, and London) and Alberta (city of Calgary), Canada. We acquired anonymized electronic medical record data from each hospital.

## Measures

### Patient characteristics

We extracted each patient’s age; sex; language preference; history of dementia, delirium, psychotic disorder (schizophrenia, bipolar disorder, schizoaffective disorder, psychotic depression, or psychosis not otherwise specified); receipt of palliative care; COVID-19 positivity; receipt of COVID-19 test; dates of hospital admission and discharge; and intensive care unit admission and discharge, where applicable (ICD-10 codes in S1 Table). We defined COVID-19 positivity as any positive COVID-19 test during a patient’s hospitalization and receipt of a COVID-19 test as any documentation in the patient visit record of a COVID-19 test being administered during a hospitalization. These measures were only calculated among individuals who were in-hospital during the COVID-19 pandemic (March 2020 onwards).

### Chemical restraint use

We extracted medication orders for antipsychotics (haloperidol, risperidone, olanzapine, quetiapine, methotrimeprazine, and loxapine), benzodiazepines (lorazepam, diazepam, and midazolam), and trazodone, which were prescribed regularly or on as-needed basis [22, 23]. Medication start and stop dates were used to determine whether a patient was being prescribed a chemical restraint on each day of their hospitalization.

### Physical restraint use

Physical restraints were soft or hard (e.g., wrist restraints, lap belt, ankle restraints, mittens, side rails). Availability of physical restraint data varied by site [5, 6]. All Alberta sites and two Ontario sites had data on physical restraints orders, three Ontario sites had data on physical restraint applications, and one Ontario site had no physical restraint data. Restraint orders and applications were examined separately. Application/order start and stop dates were used to determine whether a patient was exposed to a physical restraint on each day of hospitalization.

## Statistical analysis

### Time series analysis

We calculated the daily proportion of inpatients with restraint use between November 1, 2019, and May 31, 2020 (June 2020 was excluded to allow for a 30-day hospital stay) [24]. We modeled the daily proportion of restraint use using linear regression models with autoregressive terms and random intercepts included on the hospital level. Models were fit separately by type of restraint and province. The number of autoregressive terms was guided by the examination of hospital-level partial auto-correlation function plots. We employed restricted cubic splines with knots on the first day of each month to flexibly model changes in restraint proportion over time [25]. We fit unadjusted models and models adjusted for the prevalence of dementia and psychotic disorders. For visualization of trends, we plotted the proportion of restraint use by type, province, and hospital site, with the cubic spline fits [24].

We compared restraint use at the onset of the pandemic to pre-pandemic periods using adjacent time periods anchored by the date on which each province restricted non-urgent hospital admissions. This choice was based in our hypothesis that it was the expectation of an imminently overwhelmed system and the impact of public health restrictions, rather than an actual influx of COVID-19 patients, that led to an increase in restraint use. We defined four consecutive two-week periods starting at the date that non-urgent admissions were restricted (Alberta–March 18, 2020; Ontario–March 15, 2020) [26]. Preceding these pandemic periods, we defined a two-week washout period during which public transmission of COVID-19 had begun but there were not yet public health restrictions, and a four-week pre-pandemic period during which there was minimal to no COVID-19 transmission. We compared the observed proportion, unadjusted proportion, and adjusted proportion of chemical and physical restraint use during each two-week period to the pre-pandemic period. All comparisons were made using a parametric bootstrapping approach with 5000 replicates [27]. All analyses were completed in R, version 3.6.2. Modelling was performed with the package *nlme* and data visualization with the package *ggplot2* [28, 29]. Parameters for the parametric bootstrap and *nlme* code are available in S1 Text and S3 Table.

### Between-hospital variability

We converted the time series into a patient-level cohort with binary indicators of restraint use at any time during the hospital stay and calculated the hospital-level intraclass correlation coefficient (ICC) and median odds ratio (OR) using mixed-effect logistic regression, adjusting for available patient characteristics. We examined the ICC and median OR overall and compared the measures before and during the pandemic.

### Sensitivity analyses

We examined the effect of excluding (1) medications ordered on an as-needed basis and (2) patients admitted to intensive care units. We also used the patient-level cohort to visualize trends in the proportion of patients who ever received a chemical or physician restraint based on their hospital admission date.

### Ethics approval

For hospitals in Ontario, Canada, we obtained ethics approval through Clinical Trials Ontario (project #3300). For hospitals in Alberta, Canada, we obtained ethics approval through the University of Calgary Research Ethics Board. Each research ethics board waived the requirement for obtaining informed consent to access anonymized medical record data included in this study.

## Results

**Fig 1:**
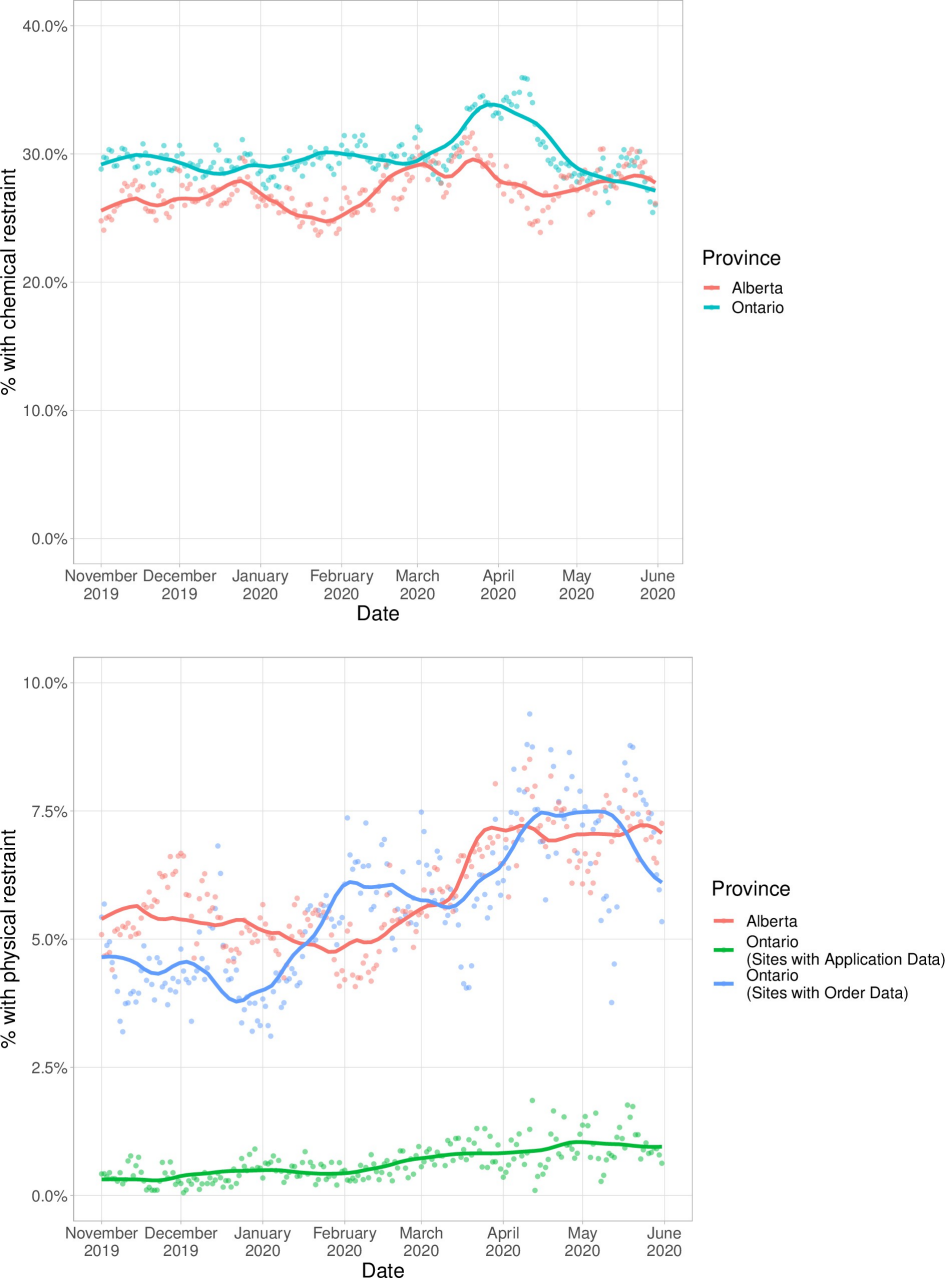
a. Proportion of older adults (≥65) admitted to acute care hospitals who were ordered chemical restraints in Alberta and Ontario, November 2019 to May 2020. b. Proportion of older adults (≥65) admitted to acute care hospitals who were ordered physical restraints in Alberta and Ontario, November 2019 to May 2020.

There were 71,004 hospitalizations among patients aged 65 years or older across 10 hospitals during the study period. Nearly two-thirds (65%) of hospitalizations were in Ontario. The median population age was 76 (q1-q3, 70–73), 48% of patients were female, and 93% of patients spoke English (Table 1, S2 Table). Chemical restraint use was uniformly more common than physical restraints in Alberta and Ontario (Fig 1A and 1B). Overall use of chemical and physical restraints was similar in both provinces.

**Table 1:** Characteristics of older adults (≥65 years) admitted to acute care hospitals in Alberta and Ontario, November 2019 to June 2020.

Characteristic	Alberta n = 24,642	Ontario N = 46,362
Female sex, n (%)	11,7826 (48)	22,201 (48)
Age, years, (median, q1, q3)	76 (70, 84)	76 (70, 83)
Non-English speaker*, n (%)	1,715 (7)	3,219 (7)
Diagnosis of dementia, n (%)	1,109 (5)	1,983 (4)
Diagnosis of delirium, n (%)	44 (<1)	3,096 (7)
Diagnosis of psychotic disorder^**†**^, n (%)	86 (<1)	392 (1)
Received palliative care^‡^, n (%)	^‡^	2,248 (6)
COVID test administered^§^, n (%)	^§^	9,021 (63)
COVID test positive^||^, n (%)	^||^	600 (3)

* One Ontario site missing, denominator = 68,284

† Schizophrenia, bipolar disorder, schizoaffective disorder, psychotic depression, or psychosis

‡ All Alberta sites and one Ontario site missing, denominator = 36,225

§ All Alberta sites and one Ontario site missing, only records during COVID-19 period included, denominator = 14,368

|| All Alberta sites missing, only records during COVID-19 period included denominator = 18,501

Abbreviations: number (n), percentage (%)

### Time series analysis

#### Chemical restraint orders

The use of one autoregressive term for chemical restraint use in Ontario, and three autoregressive terms for the other outcomes best fit the partial autocorrelation function plots (S1 Fig). In Ontario, the average unadjusted proportion of older adults ordered chemical restraints during the pre-pandemic period was 29.5% (Table 2). Chemical restraint use peaked in the fifth and sixth weeks following restriction of non-urgent admissions, with an unadjusted increase of 3.9% (95% CI, 2.0%, 6.0%) and adjusted increase of 3.7% (1.6%,5.9%) compared to the pre-pandemic period. In Alberta, the average unadjusted proportion of older adults ordered chemical restraints during the pre-pandemic period was 27.5%. There were no significant changes in chemical restraint use in Alberta across time periods.

**Table 2:** In-hospital use of chemical and physical restraints among older adults at the onset of the COVID-19 pandemic.

Time Period	Weekly Covid-19 Incidence per 100,000	Observed proportion with restraint	Unadjusted proportion with restraint	Unadjusted difference to pre-pandemic (95% CI)	Adjusted * proportion with restraint	Adjusted* difference to pre-pandemic (95% CI)
**Alberta Chemical Restraints Orders**						
Pre-pandemic (Feb5-Mar3)	0.0	27.4%	27.5%		21.2%	
Washout (Mar4-Mar17)	1.2	30.0%	29.2%	1.7% (-0.4%, 3.8%)	22.1%	0.9% (-0.9%, 2.6%)
Pandemic Weeks 1–2 (Mar18-Apr1^†^	10.0	28.8%	28.4%	1.0% (-2.4%, 4.4%)	20.6%	-0.5% (-3.4%, 2.2%)
Pandemic Weeks 3–4 (Apr2-Apr14)	9.5	26.9%	27.7%	0.3% (-3.5%, 4.2%)	19.9%	-1.3% (-4.5%, 1.8%)
Pandemic Weeks 5–6 (Apr15-Apr28)	34.6	26.4%	27.6%	0.1% (-3.7%, 3.9%)	20.2%	-0.9% (-4.0%, 2.1%)
Pandemic Weeks 7–8 (Apr29-May12)	16.1	27.7%	27.6%	0.1% (-3.8%, 3.9%)	20.7%	-0.5% (-3.4%, 2.5%)
**Alberta Physical Restraints Orders**						
Pre-pandemic (Feb5-Mar3)	0.0	5.1%	5.2%		3.3%	
Washout (Mar4-Mar17)	1.2	6.1%	6.1%	0.9% (0.0%, 1.8%)	3.8%	0.6% (-0.4%, 1.5%)
Pandemic Weeks 1–2 (Mar18-Apr1) ^†^	10.0	6.7%	6.8%	1.7% (0.3%, 3.1%)	4.3%	1.0% (-0.6%, 2.6%)
Pandemic Weeks 3–4 (Apr2-Apr14)	9.5	7.3%	7.2%	2.0% (0.4%, 3.5%)	4.5%	1.3% (-0.5%, 3.1%)
Pandemic Weeks 5–6 (Apr15-Apr28)	34.6	7.2%	7.1%	1.9% (0.4%, 3.5%)	4.6%	1.4% (-0.4%, 3.1%)
Pandemic Weeks 7–8 (Apr29-May12)	16.1	6.7%	7.0%	1.8% (0.3%, 3.4%)	4.7%	1.4% (-0.3%, 3.1%)
**Ontario Chemical Restraints Orders**						
Pre-pandemic (Feb2-Feb29)	0.0	30.0%	29.5%		27.1%	
Washout (Mar1-Mar14)	0.3	29.5%	30.3%	0.8% (-0.4%, 2.1%)	27.7%	0.6% (-0.7%, 1.9%)
Pandemic Weeks 1–2 (Mar15-Mar28) ^†^	3.5	32.8%	32.7%	3.2% (1.4%, 5.0%)	29.9%	2.9% (1.1%, 4.8%)
Pandemic Weeks 3–4 (Mar29-Apr11)	18.7	34.1%	33.4%	3.9% (2.0%, 6.0%)	30.8%	3.7% (1.6%, 5.9%)
Pandemic Weeks 5–6 (Apr12-Apr25)	25.0	31.1%	31.5%	2.0% (0.1%, 3.8%)	29.4%	2.4% (0.4%, 4.4%)
Pandemic Weeks 7–8 (Apr26-May9)	20.3	28.3%	29.1%	-0.5% (-2.3%, 1.5%)	27.6%	0.6% (-1.5%, 2.6%)
**Ontario Physical Restraint Orders**						
Pre-pandemic (Feb2-Feb29)	0.0	6.1%	5.9%		5.9%	
Washout (Mar1-Mar14)	0.3	6.2%	5.7%	-0.2% (-1.3%, 0.9%)	5.7%	-0.2% (-1.4%, 1.0%)
Pandemic Weeks 1–2 (Mar15-Mar28) ^†^	3.5	6.3%	6.1%	0.2% (-1.4%, 1.7%)	6.1%	0.2% (-1.6%, 1.9%)
Pandemic Weeks 3–4 (Mar29-Apr11)	18.7	7.1%	6.7%	0.8% (-1.1%, 2.6%)	6.9%	1.0% (-1.0%, 3.0%)
Pandemic Weeks 5–6 (Apr12-Apr25)	25.0	7.4%	7.4%	1.4% (-0.2%, 3.1%)	7.9%	2.0% (0.1%, 3.9%)
Pandemic Weeks 7–8 (Apr26-May9)	20.3	7.4%	7.6%	1.6% (0.0%, 3.3%)	8.3%	2.4% (0.4%, 4.5%)
**Ontario Physical Restraint Applications**						
Pre-pandemic (Feb2-Feb29)	0.0	0.4%	0.6%		0.6%	
Washout (Mar1-Mar14)	0.3	0.6%	0.8%	0.2% (-0.2%, 0.6%)	0.7%	0.2% (-0.2%, 0.6%)
Pandemic Weeks 1–2 (Mar15-Mar28) ^†^	3.5	0.7%	0.8%	0.2% (-0.3%, 0.7%)	0.8%	0.2% (-0.3%, 0.8%)
Pandemic Weeks 3–4 (Mar29-Apr11)	18.7	0.7%	0.8%	0.3% (-0.3%, 0.9%)	0.9%	0.3% (-0.3%, 1.0%)
Pandemic Weeks 5–6 (Apr12-Apr25)	25.0	0.7%	1.0%	0.4% (-0.2%, 0.9%)	1.0%	0.4% (-0.2%, 1.0%)
Pandemic Weeks 7–8 (Apr26-May9)	20.3	0.8%	1.0%	0.5% (-0.1%, 1.0%)	1.1%	0.5% (-0.1%, 1.1%)

CI: Confidence interval

*Adjusted for prevalence of dementia and psychotic disorders

†Pandemic week 1 anchored to start of restriction of elective/non-urgent acute care hospital admissions. Alberta: March 18, Ontario: March 15

### Physical restraint orders

In Ontario, the average unadjusted proportion of older adults ordered physical restraints during the pre-pandemic period was 5.9% (Table 1). Physical restraint use was highest in the seventh^-^ and eighth weeks following restriction of non-urgent admissions, with an unadjusted increase of 1.6% (0.0%, 3.3%) and an adjusted increase of 2.4% (0.4%, 4.5%) compared to the pre-pandemic period. In Alberta, the average unadjusted proportion of older adults ordered physical restraints during the pre-pandemic period was 5.2%. While there were significant increases in the unadjusted proportion of physical restraint orders during the pandemic period, none of the differences were significant after adjustment for known diagnoses of dementia and psychotic disorders.

### Physical restraint applications

In Ontario, the average unadjusted proportion of older adults who had physical restraints applied during the pre-pandemic period was 0.6%. While there was a trend of increasing physical restraint applications across the pandemic similar to physical restraint orders in Ontario, no differences were significant.

### Assessing between-hospital variability

**Fig 2:**
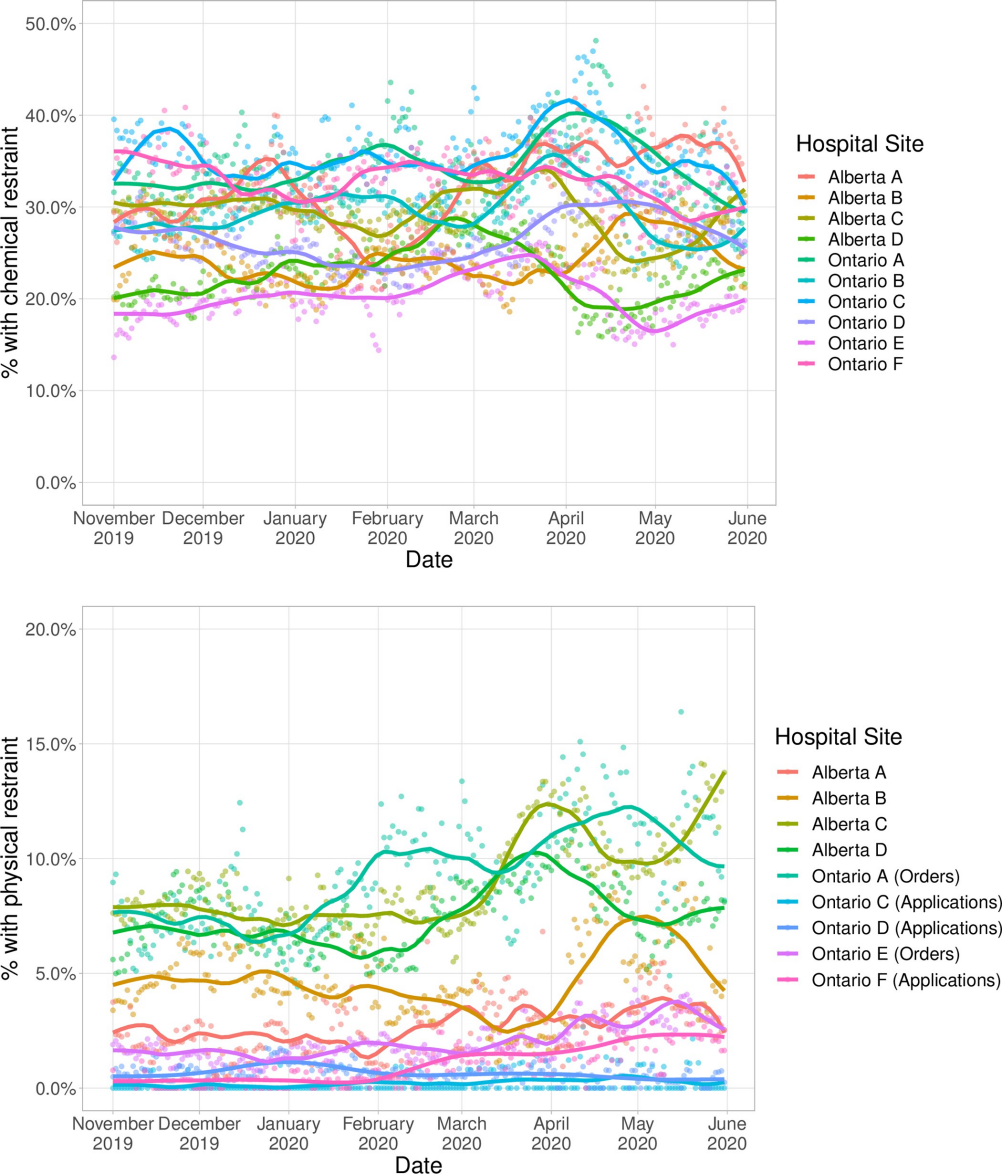
a. Proportion of older adults (≥65) admitted to acute care hospitals who were ordered chemical restraints in Alberta and Ontario, November 2019 to May 2020, by hospital site. b. Proportion of older adults (≥65) admitted to acute care hospitals who were ordered chemical restraints in Alberta and Ontario, November 2019 to May 2020, by hospital site.

There was moderate between-site variability in chemical restraint use (ICC 0.041 and median OR 1.43); variability was higher for physical restraint use (ICC 0.011 and median OR 1.83) (Fig 2A and 2B). For chemical restraints, variability was slightly higher during the pandemic period (ICC: 0.047, MOR: 1.47) than before the pandemic (ICC: 0.037, MOR: 1.40). For physical restraints, variability was lower during the pandemic period (0.087, MOR: 1.70) compared to before the pandemic period (ICC 0.146, MOR: 2.04)

### Sensitivity analyses

**Fig 3:**
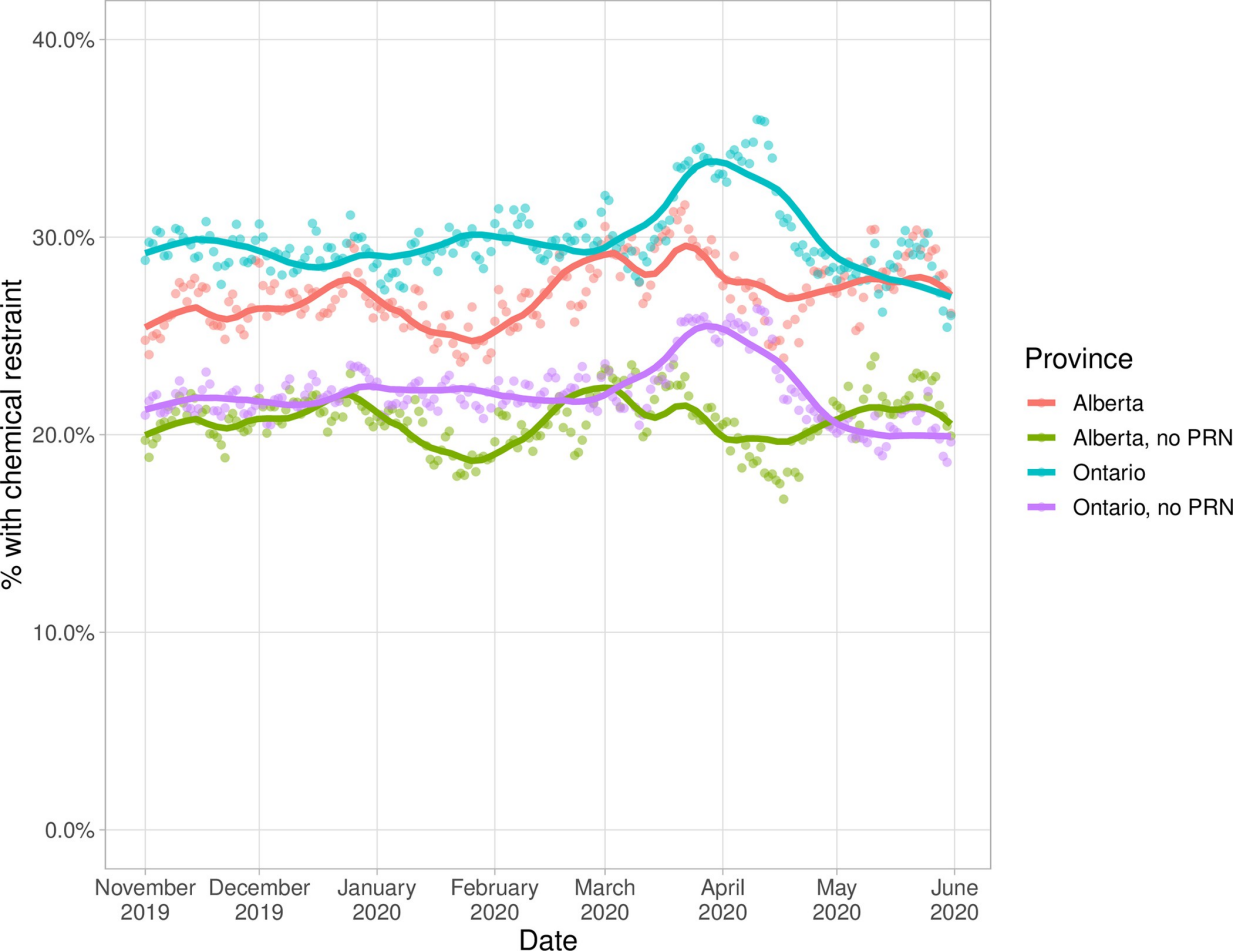
Proportion of older adults (≥65) admitted to acute care hospitals who were ordered chemical restraints in Alberta and Ontario, November 2019 to May 2020, with and without as needed medications. Abbreviation: PRN = as-needed.

Sensitivity analyses produced similar results to the main analyses for chemical restraints but with overall lower levels of restraint usage (S3 and S4 Tables, Fig 3). For physical restraints, removing patients admitted to the ICU curtailed the adjusted pandemic increase from 2.4% in the main analysis to 0.9% in the sensitivity analysis (S5 Table). The plot of the proportion of patients who ever received chemical or physical restraints looks similar to the main analysis (S2 Fig).

## Discussion

We conducted a retrospective cohort study and time series analysis describing chemical and physical restraint use among older adults admitted to acute care hospitals in Ontario and Alberta, Canada, immediately preceding and following the onset of the COVID-19 pandemic. Compared to the pre-COVID-19 pandemic period, both chemical and physical restraint use among older adults increased in Ontario acute care hospitals, but there was no significant change in restraint use of either type in Alberta acute care hospitals. There was moderate variation between hospital sites in chemical restraint use and high variation in physical restraint use. Chemical restraint use variability was higher and physical restraint use variability was lower after the onset of the COVID-19 pandemic. Our findings suggest there is an urgent need to understand differences in chemical and physical restraint use between regions and hospitals.

The increased chemical restraint use within Ontario hospitals that we observed is similar to reported increases in nursing homes [13]. Our finding of increased physical restraint use is novel within a Canadian context, though similar results were reported in patients with dementia admitted to acute care hospitals in Japan during the COVID-19 pandemic [30]. While we cannot conclusively determine the reasons behind this increased restraint use, one likely contributor was restrictive hospital visitation policies that increased social isolation and reduced opportunities for caregiver advocacy [31]. Other potential contributors include staffing shortages and strains related to caring for patients with COVID-19. However, Alberta also implemented a visitor restriction policy but did not experience an increase in restraint use. This suggests there are other differences between hospitals in Alberta and Ontario, perhaps relating to different institutional practices and culture [32, 33]. It could also be related to previous quality improvement work in Alberta [3]. In a stepped wedge trial, local champions in nursing leadership, education and training of physicians and unit nurses, and implementation of least restraint rounds reduced the rate of physical restraint use in four acute care medical units in Calgary, Alberta [3].

Significant between-hospital differences in chemical and physical restraint use suggest that there is substantial practice variation and opportunities to improve best practices. Local barriers such as clinician knowledge, skills, social/professional role and identity, and environmental context and resources need to be studied further to inform efforts to improving the appropriateness of restraint use [34]. Quality improvement initiatives can support the implementation of recommendations to reduce inappropriate restraint orders in acute care hospitals and improve the quality of acute care for older adults. For example, monitoring-benchmarking and multidisciplinary education reduced the rate of benzodiazepine prescribing in five Swiss teaching hospitals [35]. From a policy perspective, hospitals need to consistently measure orders for chemical and physical restraints to monitor changes in their use. The sustainability of initiatives that influence and monitor practice patterns will be limited in hospitals where orders cannot be quickly and accurately tracked.

Our study had limitations. First, we likely underestimated physical restraint use because some restraint types are used without a medical order (e.g., bed rails, tilt chairs). Second, we could not adjust for seasonality in our analyses; however, we did not expect significant seasonality in restraint use. Third, the prevalence of dementia and delirium was likely underestimated because these diagnoses are underreported in health administrative data, but we would not expect differential misclassification by restraint type or time period (that is, pre-pandemic versus pandemic periods) [36]. Lastly, the 10 hospital sites in our study cohort are located in large urban centres, which means our findings may not generalize to all community, rural and remote hospital sites.

In conclusion, chemical and physical restraint use among older adults across acute care hospitals was impacted by the COVID-19 pandemic in Ontario but not Alberta, Canada. Substantial between-hospital variability in chemical and physical restraint use suggests a larger problem: the need to reduce inappropriate orders for chemical and physical restraints in acute care hospitals and improve the quality of acute care for older adults. Future research must help us to better understand these practice variations so that clinicians, researchers, policy makers and other stakeholders can work together to implement evidence-informed interventions that support the appropriate use of chemical and physical restraints for older adults admitted to acute care hospitals.

## Supporting information

S1 Table ICD-10 definitions of conditions used in analysis. (PDF)

S2 Table Descriptive characteristics of included acute care hospitals. (PDF)

S3 Table Parameters used in parametric bootstrap. Distribution: Gaussian. Number of replicates = 5000.
(PDF)

S4 Table In-hospital use of chemical restraints among older adults at the onset of the COVID-19 pandemic, as-needed medications excluded. 1. Adjusted for prevalence of dementia and psychotic disorders. 2. Pandemic week 1 anchored to start of restriction of elective/non-urgent acute care hospital admissions. Alberta: March 18, Ontario: March 15.
(PDF)

S5 Table In-hospital use of chemical and physical restraints among older adults at the onset of the COVID-19 pandemic, ICU patients excluded. 1. Adjusted for prevalence of dementia and psychotic disorders. 2. Pandemic week 1 anchored to start of restriction of elective/non-urgent acute care hospital admissions. Alberta: March 18, Ontario: March 15.
(PDF)

S1 Text *nlme* code for autoregressive linear mixed models. (PDF)

S1 Fig Partial autocorrelation function plots. (PDF)

S2 Fig Proportion of older adults (≥65) admitted to acute care hospitals who were ever ordered chemical or physical restraints in Alberta and Ontario, by day of hospital admission, November 2019 to May 2020. (PDF)
