# Supplementary material for: Chemical and physical restraint use during acute care hospitalization of older adults: A retrospective cohort study and time series analysis
Source: PLoS One. 2022 Oct 26;17(10):e0276504. doi: 10.1371/journal.pone.0276504 (PMC9604990; doi:10.1371/journal.pone.0276504)
Supplement: S1 Table — (PDF) [file pone.0276504.s001.pdf]

S1 Table. ICD-10 definitions of conditions used in analysis

Condition	ICD-10 definition
Dementia	F00 – F03
Delirium	F05*, F10-19 (4th digit of .4 only)
Schizophrenia	F20*, F21, F23.1, F23.2
Bipolar Disorder	F31*
Schizoaffective Disorder	F25*
Psychotic Depression	F32.3, F33.3
Psychosis	F23*, F28, F29, F10-19 (4th digit of .5 and .7 only)
