# Supplementary material for: Chemical and physical restraint use during acute care hospitalization of older adults: A retrospective cohort study and time series analysis
Source: PLoS One. 2022 Oct 26;17(10):e0276504. doi: 10.1371/journal.pone.0276504 (PMC9604990; doi:10.1371/journal.pone.0276504)
Supplement: S2 Table — (PDF) [file pone.0276504.s002.pdf]

S2 Table. Descriptive characteristics of included acute care hospitals

Hospital	Location (City, province)	Bed size	Rural or urban
1	Toronto, Ontario	1286	Urban
2	Toronto, Ontario	463	Urban
3	Toronto, Ontario	442	Urban
4	Hamilton, Ontario	865	Urban
5	Hamilton, Ontario	777	Urban
6	London, Ontario	977	Urban
7	Calgary, Alberta	1100+	Urban
8	Calgary, Alberta	600+	Urban
9	Calgary, Alberta	650+	Urban
10	Calgary, Alberta	269	Urban
