# Supplementary material for: Chemical and physical restraint use during acute care hospitalization of older adults: A retrospective cohort study and time series analysis
Source: PLoS One. 2022 Oct 26;17(10):e0276504. doi: 10.1371/journal.pone.0276504 (PMC9604990; doi:10.1371/journal.pone.0276504)
Supplement: S3 Table — Distribution: Gaussian. Number of replicates = 5000. (PDF) [file pone.0276504.s003.pdf]

S3 Table. Parameters used in parametric bootstrap

	Ontario			Alberta	
**Fixed effects**	Chemical Restraints	Physical Restraint Orders	Physical Restraint Applications	Chemical Restraints	Physical Restraint Orders
Intercept	0.237	0.28597	0.004	0.13844	0.0205
Spline1	-0.0054	-0.00386	0.000794	0.00777	-0.00145
Spline2	0.02749	0.01944	-0.00318	-0.03373	0.0051
Spline3	-0.063	-0.03915	0.0061	0.07707	-0.0053
Spline4	0.1072	0.042011	-0.00734	-0.12793	0.00354
Spline5	-0.15167	-0.01751	0.0064	0.1436	-0.00751
Spline6	0.144	-0.02263	-0.00604	-0.097807	0.00873
Dementia	0.388	0.216	0.0103	0.55188	0.1967
Psychotic Disorder	0.4928	0.501	-0.057	0.8224	0.00129
**Random Effects**					
Variance (Intercept)	0.00187	0.00229	0.00000666	0.000135	0.001178
Variance (Residual)	0.000971	0.000252	0.0000427	0.000899	0.0002583
**Autocorrelation**					
Phi1	0.858	0.8949	0.489	0.766	0.751
Phi2		-0.015	0.1375	0.0153	0.0629
Phi3		0.1157	0.1837	0.119	0.103

Distribution: Gaussian. Number of replicates = 5000
