# Supplementary material for: Chemical and physical restraint use during acute care hospitalization of older adults: A retrospective cohort study and time series analysis
Source: PLoS One. 2022 Oct 26;17(10):e0276504. doi: 10.1371/journal.pone.0276504 (PMC9604990; doi:10.1371/journal.pone.0276504)
Supplement: S4 Table — 1. Adjusted for prevalence of dementia and psychotic disorders. 2. Pandemic week 1 anchored to start of restriction of elective/non-urgent acute care hospital admissions. Alberta: March 18, Ontario: March 15. (PDF) [file pone.0276504.s004.pdf]

S4 Table. In-hospital use of chemical restraints among older adults at the onset of the COVID-19 pandemic, as-needed medications excluded

Time Period	Adjusted[1] % w/ restraint	Adjusted[1] difference to pre-pandemic (95% CI)
**Alberta Chemical Restraints**		
Pre-pandemic (Feb5-Mar3)	16.0%	
Washout (Mar4-Mar17)	16.2%	0.2% (-1.4%, 1.8%)
Pandemic Weeks 1-2 (Mar18-Apr1)[2]	14.2%	-1.8% (-4.4%, 0.8%)
Pandemic Weeks 3-4 (Apr2-Apr14)	13.5%	-2.4% (-5.4%, 0.5%)
Pandemic Weeks 5-6 (Apr15-Apr28)	14.5%	-1.4% (-4.3%, 1.3%)
Pandemic Weeks 7-8 (Apr29-May12)	15.5%	-0.5% (-3.3%, 2.3%)
**Ontario Chemical Restraints**		
Pre-pandemic (Feb2-Feb29)	19.7%	
Washout (Mar1-Mar14)	20.6%	0.9% (-0.4%, 2.1%)
Pandemic Weeks 1-2 (Mar15-Mar28)[2]	22.4%	2.7% (1.0%, 4.5%)
Pandemic Weeks 3-4 (Mar29-Apr11)	22.8%	3.1% (1.1%, 5.1%)
Pandemic Weeks 5-6 (Apr12-Apr25)	21.2%	1.5% (-0.4%, 3.3%)
Pandemic Weeks 7-8 (Apr26-May9)	19.5%	-0.2% (-2.1%, 1.7%)

1. Adjusted for prevalence of dementia and psychotic disorders
2. Pandemic week 1 anchored to start of restriction of elective/non-urgent acute care hospital admissions. Alberta: March 18, Ontario: March 15
