# Supplementary material for: Chemical and physical restraint use during acute care hospitalization of older adults: A retrospective cohort study and time series analysis
Source: PLoS One. 2022 Oct 26;17(10):e0276504. doi: 10.1371/journal.pone.0276504 (PMC9604990; doi:10.1371/journal.pone.0276504)
Supplement: S5 Table — 1. Adjusted for prevalence of dementia and psychotic disorders. 2. Pandemic week 1 anchored to start of restriction of elective/non-urgent acute care hospital admissions. Alberta: March 18, Ontario: March 15. (PDF) [file pone.0276504.s005.pdf]

S5 Table. In-hospital use of chemical and physical restraints among older adults at the onset of the COVID-19 pandemic, ICU patients excluded

Time Period	Adjusted[1] proportion w/ restraint	Adjusted[1] difference to pre-pandemic (95% CI)
**Alberta Chemical Restraints**		
Pre-pandemic (Feb5-Mar3)	21.3%	
Washout (Mar4-Mar17)	22.3%	1.0% (-0.7%, 2.8%)
Pandemic Weeks 1-2 (Mar18-Apr1)[2]	20.7%	-0.7% (-3.5%, 2.2%)
Pandemic Weeks 3-4 (Apr2-Apr14)	19.8%	-1.5% (-4.8%, 1.7%)
Pandemic Weeks 5-6 (Apr15-Apr28)	20.3%	-1.0% (-4.1%, 2.2%)
Pandemic Weeks 7-8 (Apr29-May12)	20.9%	-0.4% (-3.5%, 2.7%)
**Alberta Physical Restraints**		
Pre-pandemic (Feb5-Mar3)	3.5%	
Washout (Mar4-Mar17)	4.1%	0.6% (-0.4%, 1.5%)
Pandemic Weeks 1-2 (Mar18-Apr1)[2]	4.3%	0.8% (-0.6%, 2.3%)
Pandemic Weeks 3-4 (Apr2-Apr14)	4.6%	1.1% (-0.6%, 2.8%)
Pandemic Weeks 5-6 (Apr15-Apr28)	4.7%	1.3% (-0.3%, 2.9%)
Pandemic Weeks 7-8 (Apr29-May12)	4.8%	1.3% (-0.3%, 3.0%)
**Ontario Chemical Restraints**		
Pre-pandemic (Feb2-Feb29)	26.4%	
Washout (Mar1-Mar14)	27.0%	0.6% (-0.7%, 2.9%)
Pandemic Weeks 1-2 (Mar15-Mar28)[2]	29.2%	2.8% (1.0%, 4.5%)
Pandemic Weeks 3-4 (Mar29-Apr11)	29.9%	3.5% (1.4%, 5.6%)
Pandemic Weeks 5-6 (Apr12-Apr25)	28.4%	2.0% (0.1%, 3.9%)
Pandemic Weeks 7-8 (Apr26-May9)	26.6%	0.2% (-1.8%, 2.2%)
**Ontario Physical Restraint Orders**		
Pre-pandemic (Feb2-Feb29)	5.1%	
Washout (Mar1-Mar14)	4.8%	-0.3% (-1.4%, 0.5%)
Pandemic Weeks 1-2 (Mar15-Mar28)[2]	4.8%	-0.3% (-1.9%, 1.3%)
Pandemic Weeks 3-4 (Mar29-Apr11)	5.2%	0.1% (-1.8%, 1.9%)
Pandemic Weeks 5-6 (Apr12-Apr25)	5.7%	0.6% (-1.1%, 2.3%)
Pandemic Weeks 7-8 (Apr26-May9)	6.0%	0.9% (-0.9%, 2.7%)
**Ontario Physical Restraint Applications**		
Pre-pandemic (Feb2-Feb29)	0.6%	
Washout (Mar1-Mar14)	0.7%	0.1% (-0.2%, 0.3%)
Pandemic Weeks 1-2 (Mar15-Mar28)[2]	0.7%	0.1% (-0.2%, 0.4%)
Pandemic Weeks 3-4 (Mar29-Apr11)	0.8%	0.2% (-0.2%, 0.6%)
Pandemic Weeks 5-6 (Apr12-Apr25)	0.9%	0.3% (-0.1%, 0.6%)
Pandemic Weeks 7-8 (Apr26-May9)	0.9%	0.3% (-0.1%, 0.7%)

1. Adjusted for prevalence of dementia and psychotic disorders
2. Pandemic week 1 anchored to start of restriction of elective/non-urgent acute care hospital admissions. Alberta: March 18, Ontario: March 15
