# Supplementary material for: Chemical and physical restraint use during acute care hospitalization of older adults: A retrospective cohort study and time series analysis
Source: PLoS One. 2022 Oct 26;17(10):e0276504. doi: 10.1371/journal.pone.0276504 (PMC9604990; doi:10.1371/journal.pone.0276504)
Supplement: S1 Text — (PDF) [file pone.0276504.s006.pdf]

S1 Text. *nlme* code for autoregressive linear mixed models

```
#Chemical restraints Ontario

cr_on<-
lme(cr_p~rcspline.eval(day,nk=8,knots=c(0,30,61,92,121,150,182,212))+dem_prev+beh_prev,random=~
1|site,data=means_model%>% filter(site2=="Ontario"),correlation=corARMA(form=~day|site,p=1))

#Physical restraints orders Ontario

pr_on_o<-
lme(pr_p~rcspline.eval(day,nk=8,knots=c(0,30,61,92,121,150,182,212))+dem_prev+beh_prev,random=~
1|site,data=means_model%>% filter(site3=="Ontario\n(Sites with Order Data)" & site !=
"UHN"),correlation=corARMA(form=~day|site,p=3))

#Physical restraints applications Ontario

pr_on_a<-
lme(pr_p~rcspline.eval(day,nk=8,knots=c(0,30,61,92,121,150,182,212))+dem_prev+beh_prev,random=~
1|site,data=means_model%>% filter(site3=="Ontario\n(Sites with Application Data)" & site !=
"UHN"),correlation=corARMA(form=~day|site,p=3))

#Chemcial restraints orders Alberta

cr_ab<-
lme(cr_p~rcspline.eval(day,nk=8,knots=c(0,30,61,92,121,150,182,212))+dem_prev+beh_prev,random=~
1|site,data=means_model%>% filter(site2=="Alberta"),correlation=corARMA(form=~day|site,p=3))

#Physical restraints orders Alberta

pr_ab<-
lme(pr_p~rcspline.eval(day,nk=8,knots=c(0,30,61,92,121,150,182,212))+dem_prev+beh_prev,random=~
1|site,data=means_model%>% filter(site2=="Alberta"),correlation=corARMA(form=~day|site,p=3))

#Chemical restraints Ontario (unadjusted)

cr_on_un<-
lme(cr_p~rcspline.eval(day,nk=8,knots=c(0,30,61,92,121,150,182,212)),random=~1|site,data=means_m
odel%>% filter(site2=="Ontario"),correlation=corARMA(form=~day|site,p=1))

#Physical restraints orders Ontario (unadjusted)

pr_on_o_un<-
lme(pr_p~rcspline.eval(day,nk=8,knots=c(0,30,61,92,121,150,182,212)),random=~1|site,data=means_m
odel%>% filter(site3=="Ontario\n(Sites with Order Data)" & site
!="UHN"),correlation=corARMA(form=~day|site,p=3))

#Physical restraints applications Ontario (unadjusted)

pr_on_a_un<-
lme(pr_p~rcspline.eval(day,nk=8,knots=c(0,30,61,92,121,150,182,212)),random=~1|site,data=means_m
```

```
odel%>% filter(site3=="Ontario\n(Sites with Application Data)" & site !=
"UHN"),correlation=corARMA(form=~day|site,p=3))

#Chemcial restraints orders Alberta (unadjusted)

cr_ab_un<-
lme(cr_p~rcspline.eval(day,nk=8,knots=c(0,30,61,92,121,150,182,212)),random=~1|site,data=means_m
odel%>% filter(site2=="Alberta"),correlation=corARMA(form=~day|site,p=3))

#Physical restraints orders Alberta (unadjusted)

pr_ab_un<-
lme(pr_p~rcspline.eval(day,nk=8,knots=c(0,30,61,92,121,150,182,212)),random=~1|site,data=means_m
odel%>% filter(site2=="Alberta"),correlation=corARMA(form=~day|site,p=3))


#Chemcial restraints Ontario, no prn

cr_on_no_prn<-

lme(cr_no_prn_p~rcspline.eval(day,nk=8,knots=c(0,30,61,92,121,150,182,212))+dem_prev+beh_prev,ra
ndom=~1|site,data=means_model%>%
filter(site2=="Ontario"),correlation=corARMA(form=~day|site,p=1))

#Physical restraints Ontario, no prn

cr_ab_no_prn<-
lme(cr_no_prn_p~rcspline.eval(day,nk=8,knots=c(0,30,61,92,121,150,182,212))+dem_prev+beh_prev,ra
ndom=~1|site,data=means_model%>%
filter(site2=="Alberta"),correlation=corARMA(form=~day|site,p=3))

#Chemcial restraints Ontario, no icu

cr_on_icu<-

lme(cr_p_icu~rcspline.eval(day,nk=8,knots=c(0,30,61,92,121,150,182,212))+dem_prev+beh_prev,rando
m=~1|site,data=means_model%>% filter(site2=="Ontario"),correlation=corARMA(form=~day|site,p=1))

#Physical restraints orders Ontario, no icu

pr_on_o_icu<-

lme(pr_p_icu~rcspline.eval(day,nk=8,knots=c(0,30,61,92,121,150,182,212))+dem_prev+beh_prev,rando
m=~1|site,data=means_model%>% filter(site3=="Ontario\n(Sites with Order Data)" & site !=
"UHN"),correlation=corARMA(form=~day|site,p=3))

#Physical restraints applications Ontario, no icu


pr_on_a_icu<-
```

```
lme(pr_p_icu~rcspline.eval(day,nk=8,knots=c(0,30,61,92,121,150,182,212))+dem_prev+beh_prev,rando
m=~1|site,data=means_model%>% filter(site3=="Ontario\n(Sites with Application Data)" & site !=
"UHN"),correlation=corARMA(form=~day|site,p=3))

#Chemcial restraints applications Alberta, no icu

cr_ab_icu<-

lme(cr_p_icu~rcspline.eval(day,nk=8,knots=c(0,30,61,92,121,150,182,212))+dem_prev+beh_prev,rando
m=~1|site,data=means_model%>% filter(site2=="Alberta"),correlation=corARMA(form=~day|site,p=3))

#Physical restraints applications Alberta, no icu

pr_ab_icu<-

lme(pr_p_icu~rcspline.eval(day,nk=8,knots=c(0,30,61,92,121,150,182,212))+dem_prev+beh_prev,rando
m=~1|site,data=means_model%>% filter(site2=="Alberta"),correlation=corARMA(form=~day|site,p=3))
```
