# Supplementary material for: Chemical and physical restraint use during acute care hospitalization of older adults: A retrospective cohort study and time series analysis
Source: PLoS One. 2022 Oct 26;17(10):e0276504. doi: 10.1371/journal.pone.0276504 (PMC9604990; doi:10.1371/journal.pone.0276504)
Supplement: S1 Fig — (PDF) [file pone.0276504.s007.pdf]

S1 Figure. Partial Autocorrelation plots

<u>Chemical Restraints</u>

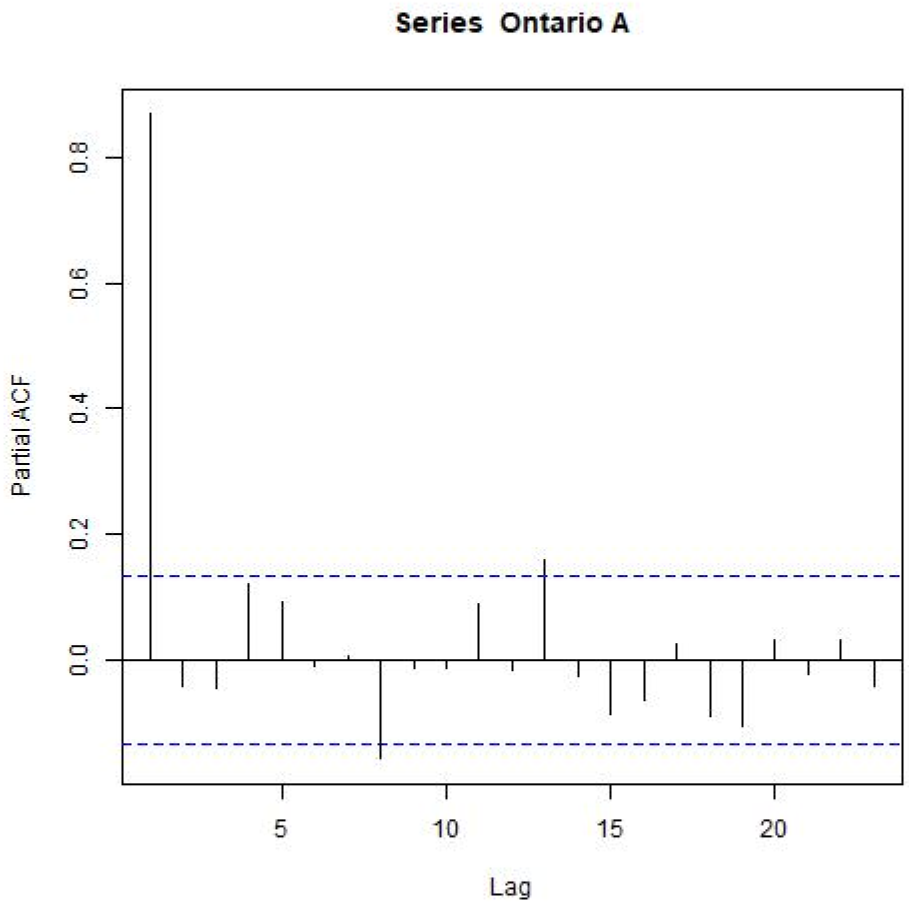

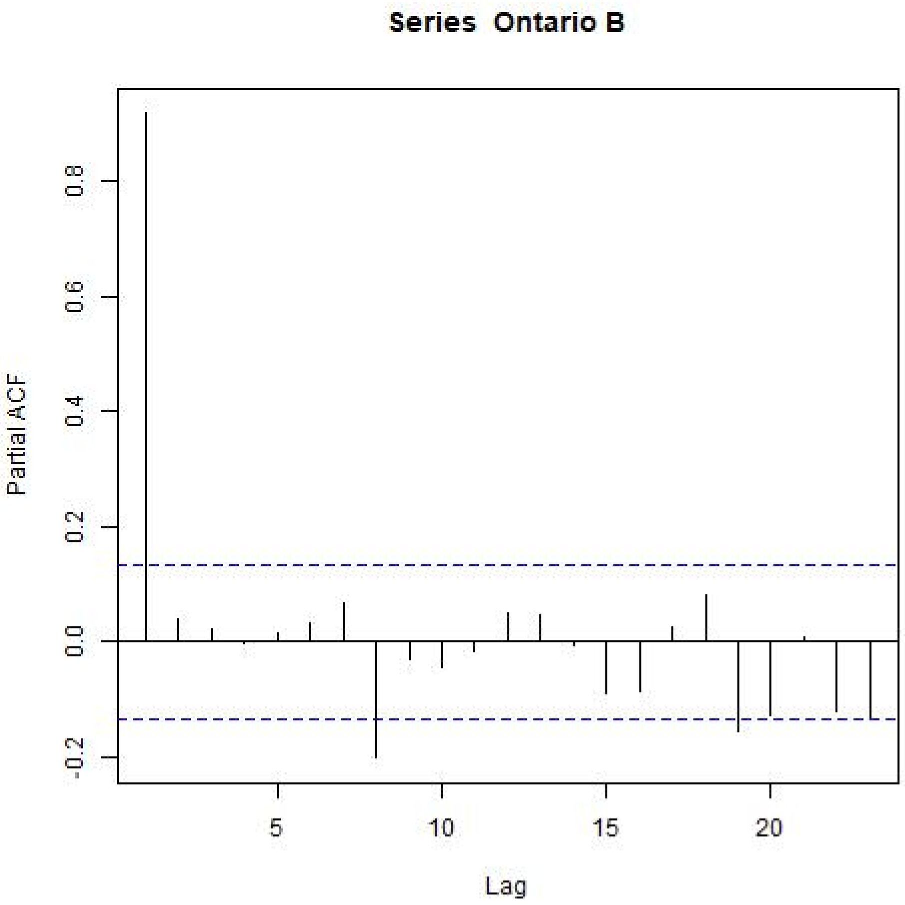
Series Ontario B
Partial ACF
0.8
0.6
0.4
0.2
0.0
-0.2
5
10
15
20
Lag

Series Ontario C

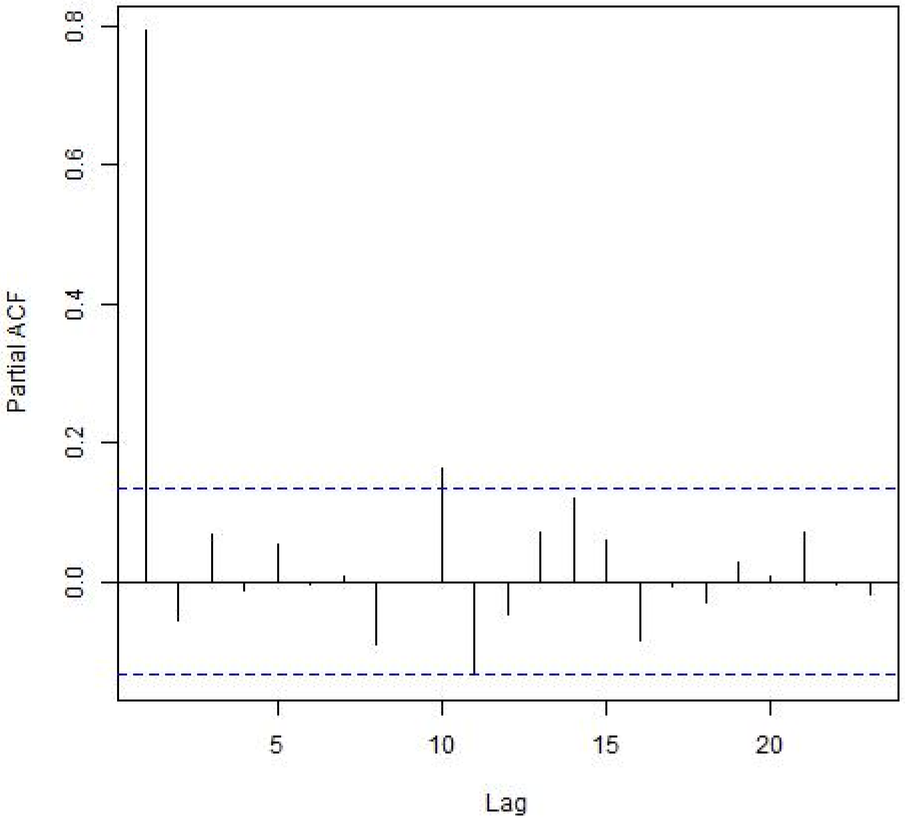

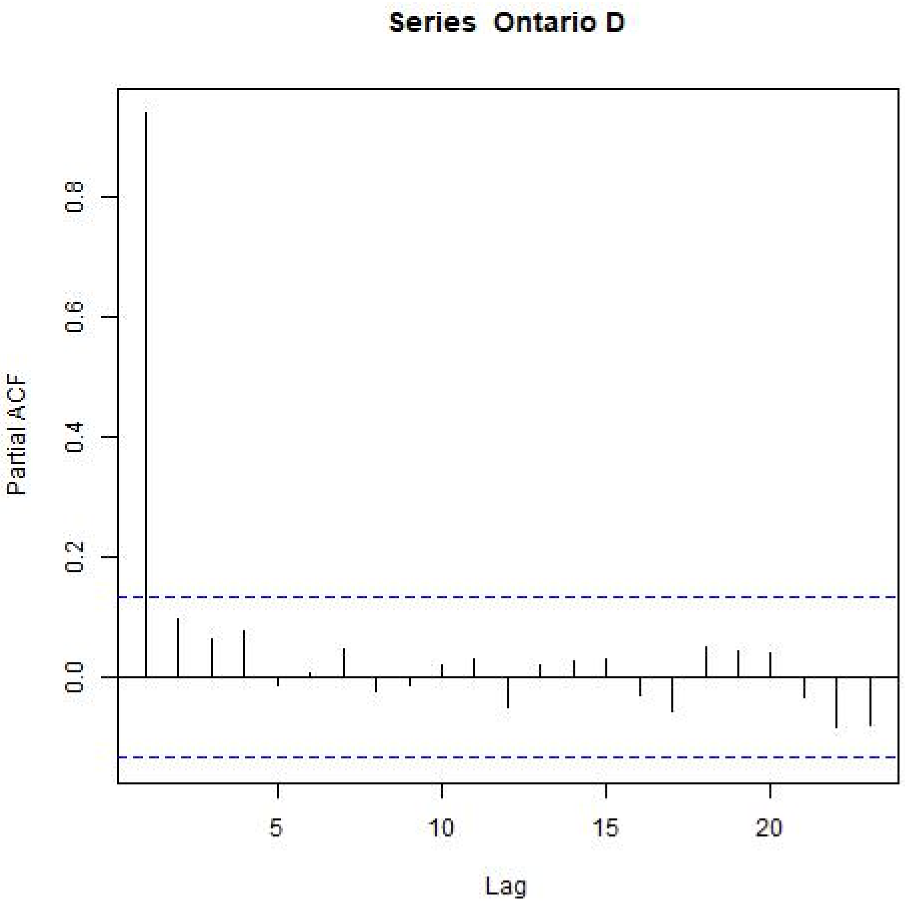

Series Ontario D
Partial ACF
0.0
0.2
0.4
0.6
0.8
5
10
15
20
Lag

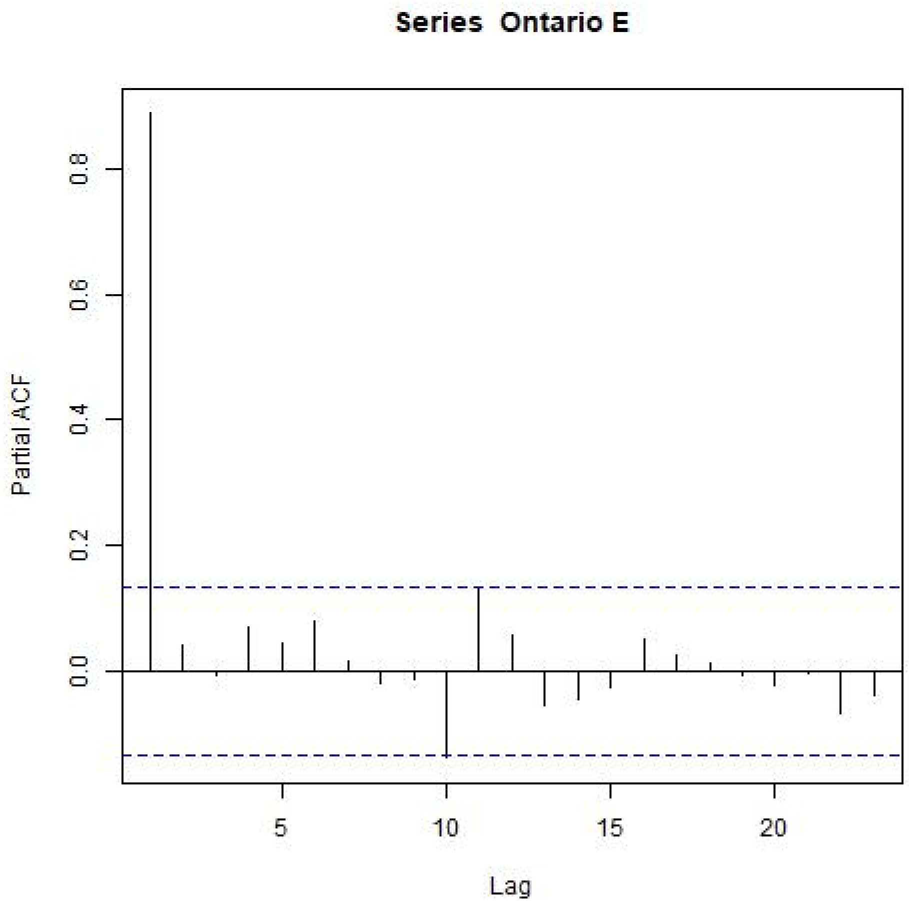

Series Ontario E
Partial ACF
0.0
0.2
0.4
0.6
0.8
5
10
15
20
Lag

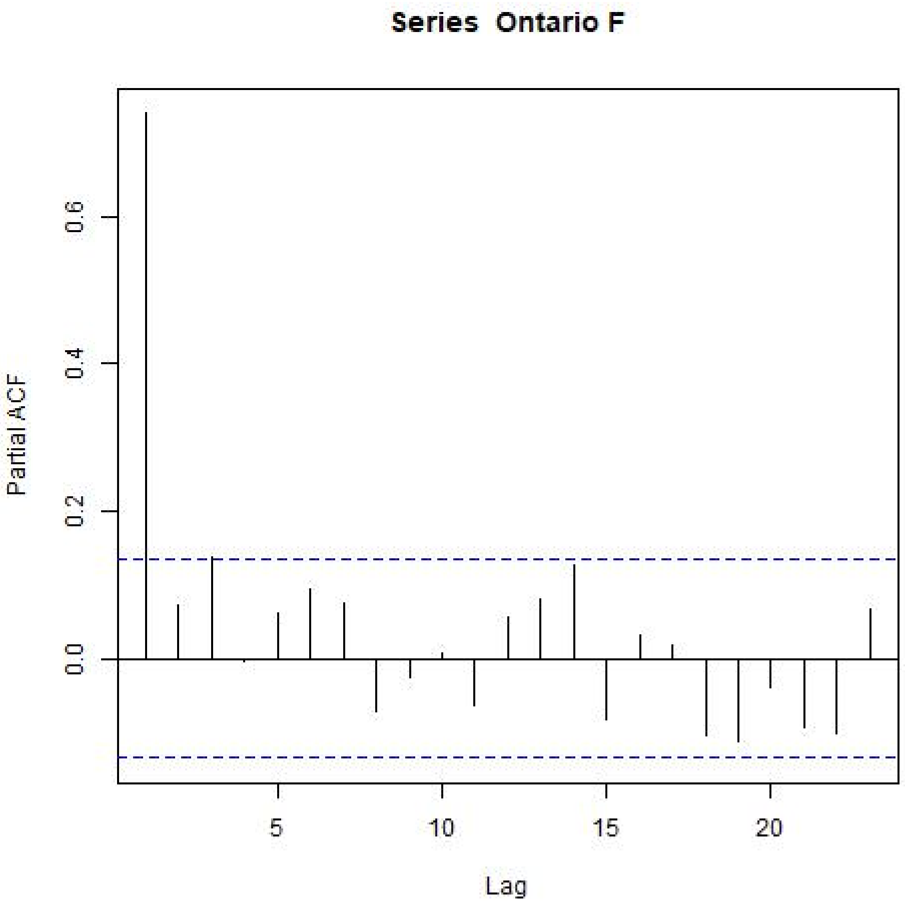

Series Ontario F
Partial ACF
0.0
0.2
0.4
0.6
5
10
15
20
Lag

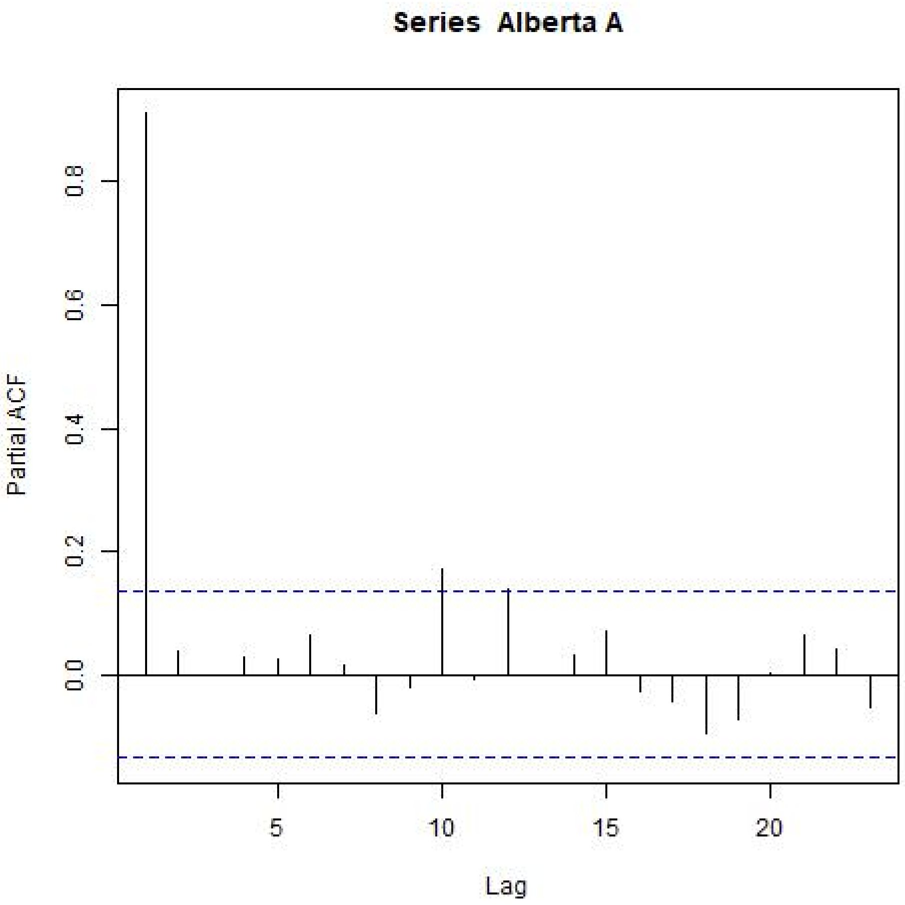

Series Alberta A
Partial ACF
0.0
0.2
0.4
0.6
0.8
5
10
15
20
Lag

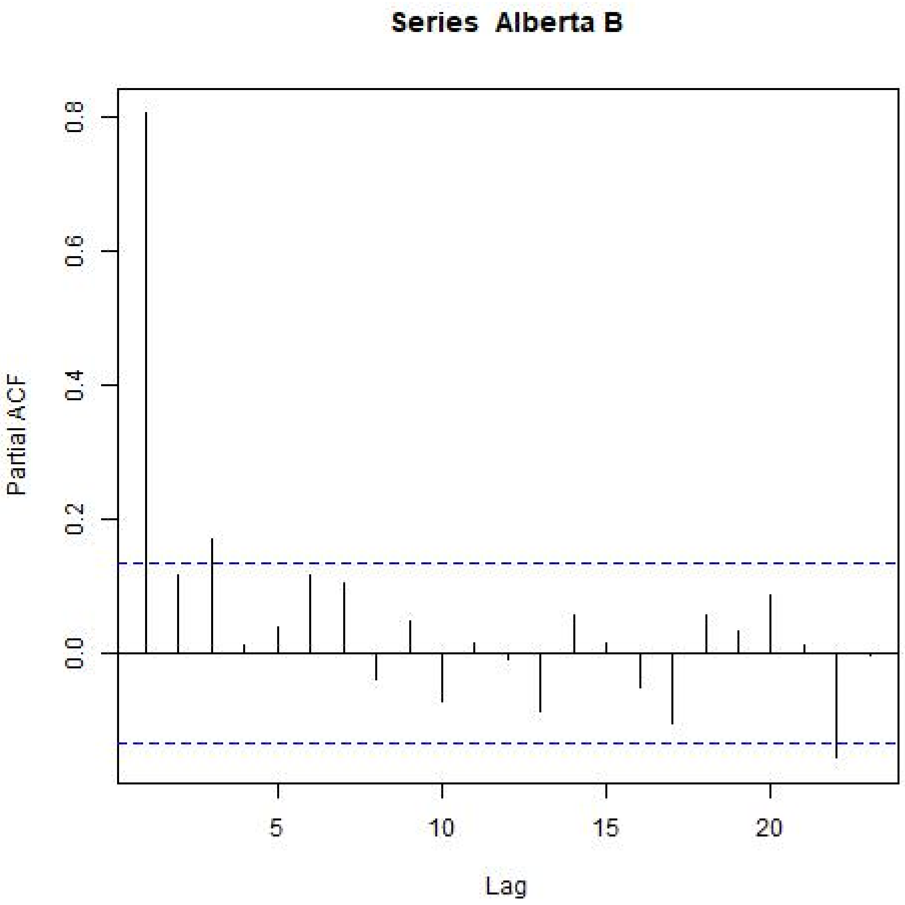
Series Alberta B
Partial ACF
0.0
0.2
0.4
0.6
0.8
5
10
15
20
Lag

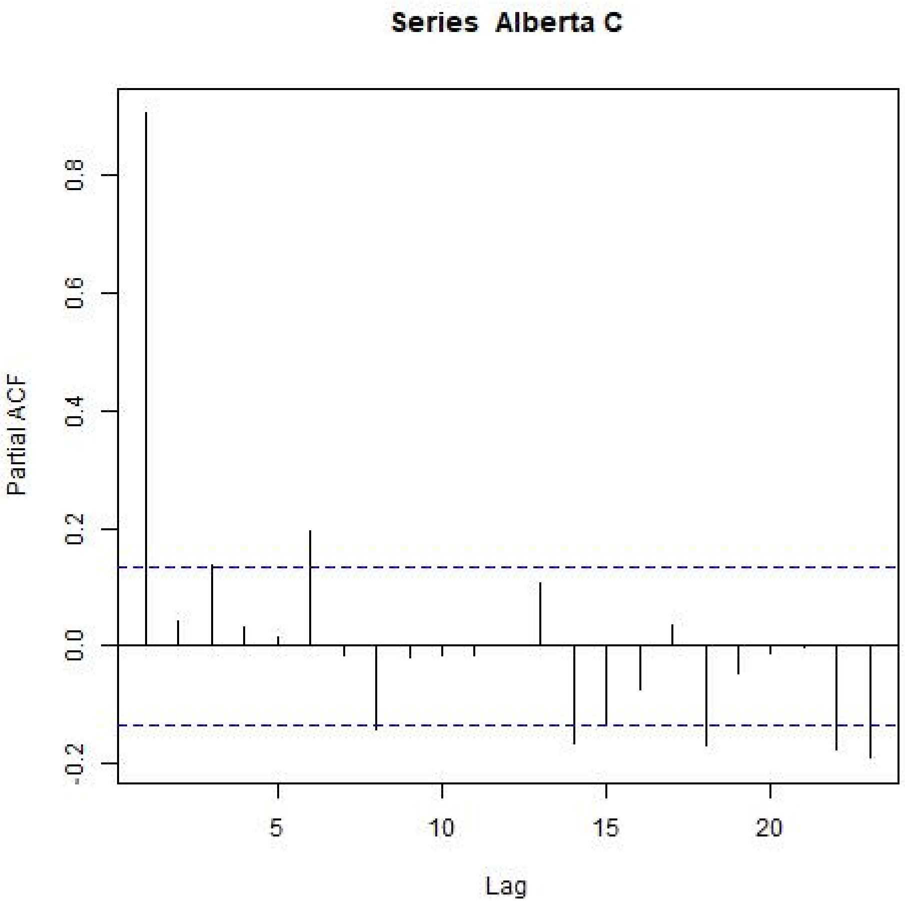

Series Alberta C
Partial ACF
-0.2
0.0
0.2
0.4
0.6
0.8
5
10
15
20
Lag

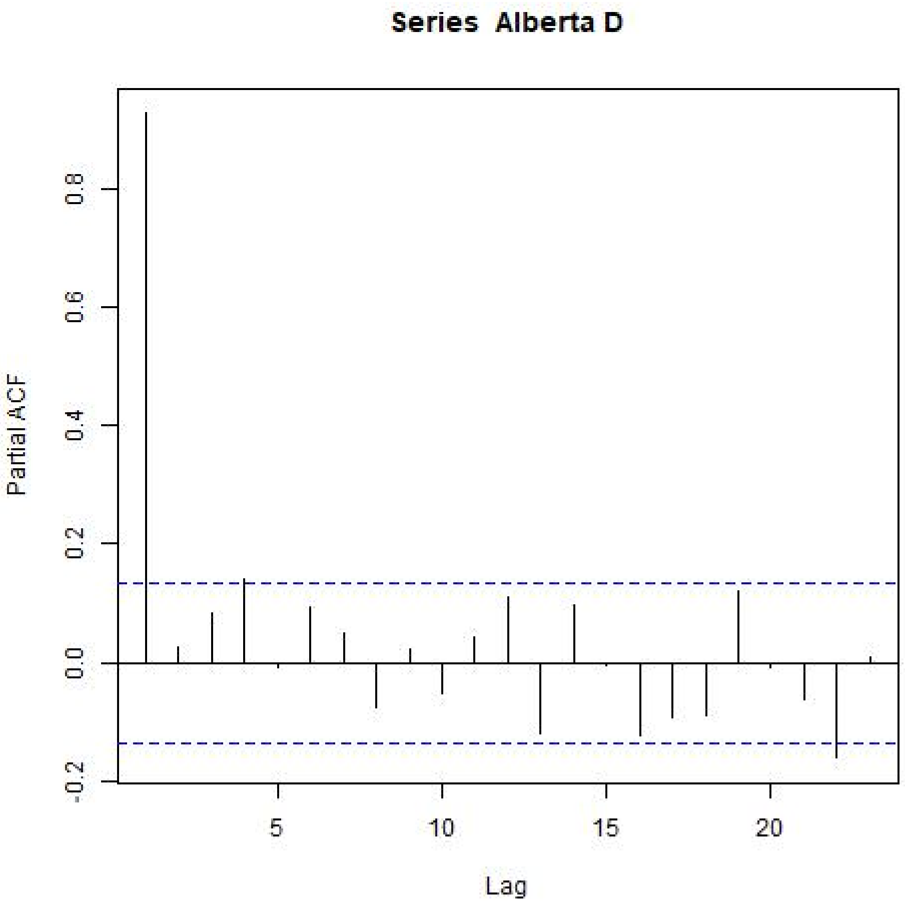
Series Alberta D
Partial ACF
0.8
0.6
0.4
0.2
0.0
-0.2
5
10
15
20
Lag

Physical Restraints

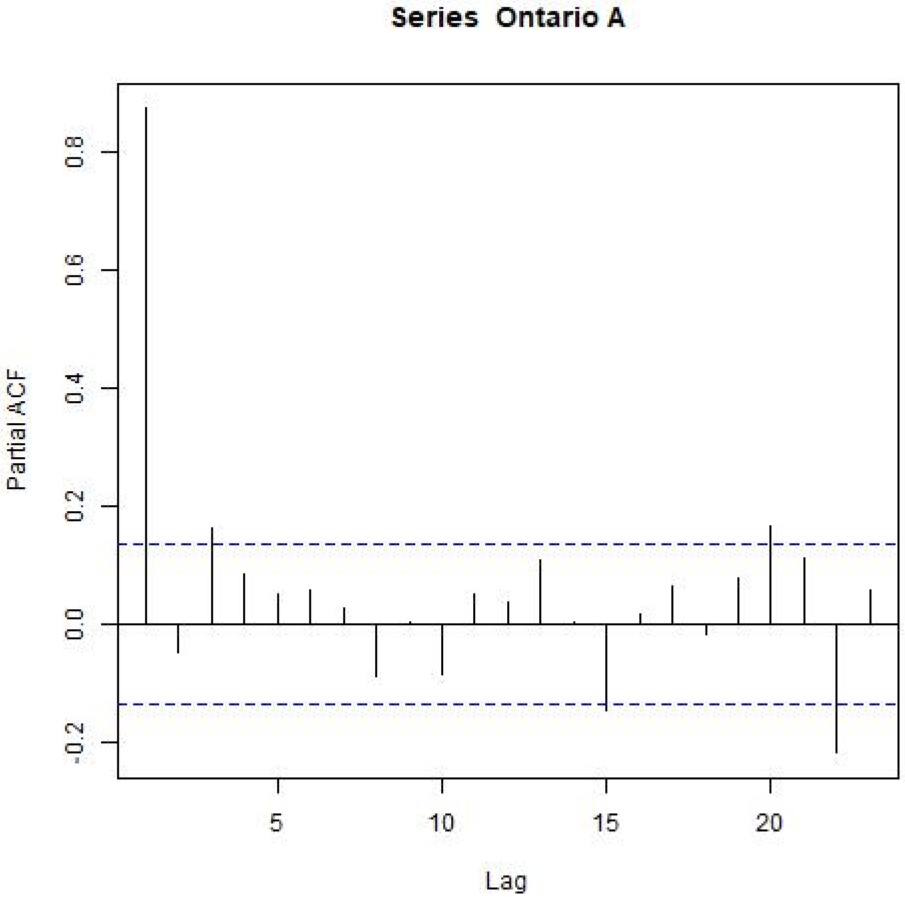

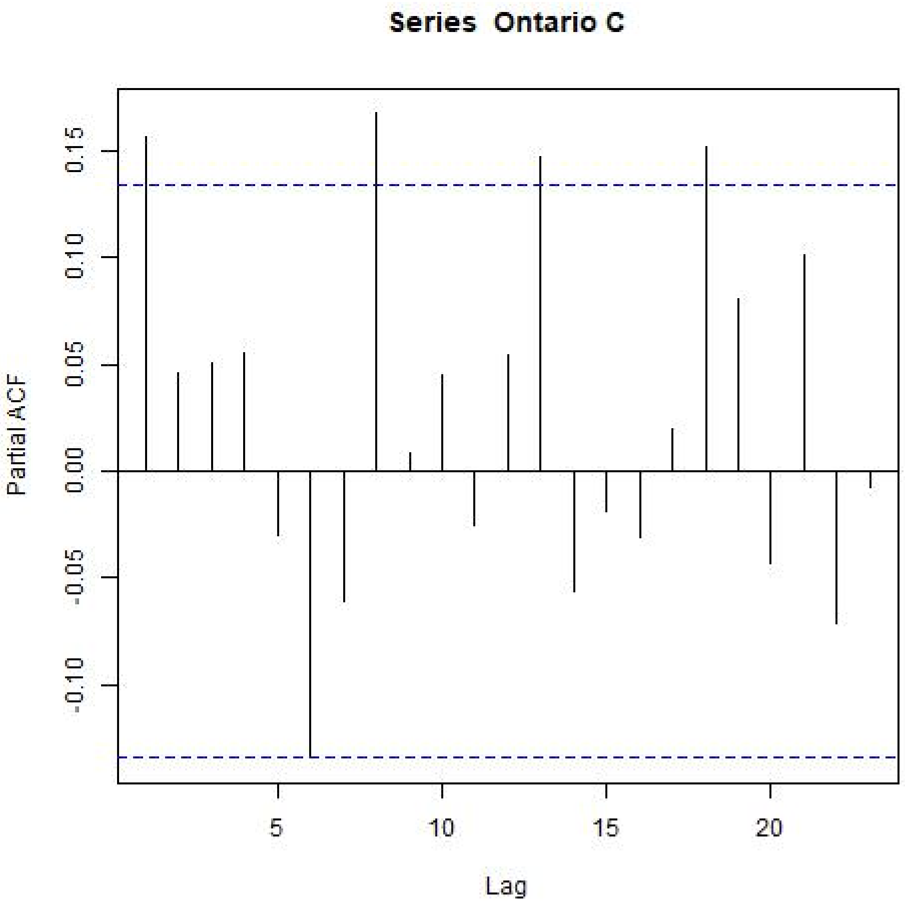
Series Ontario C
Partial ACF
0.15
0.10
0.05
0.00
-0.05
-0.10
5
10
15
20
Lag

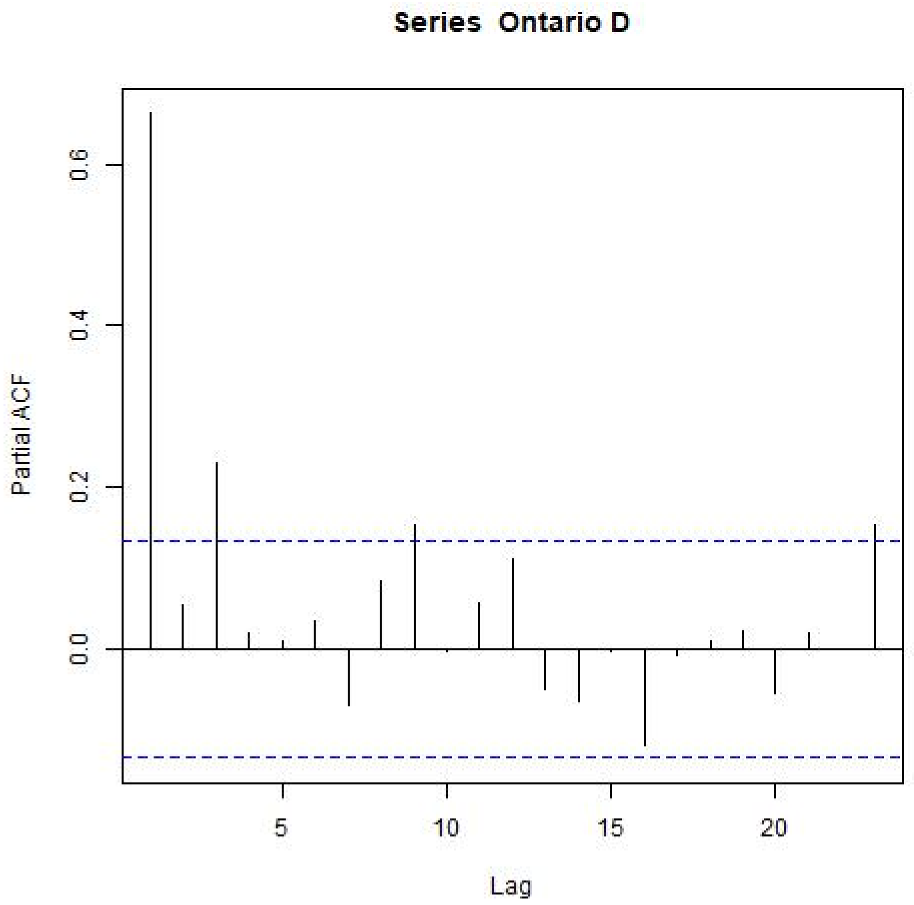

Series Ontario D
Partial ACF
0.0
0.2
0.4
0.6
5
10
15
20
Lag

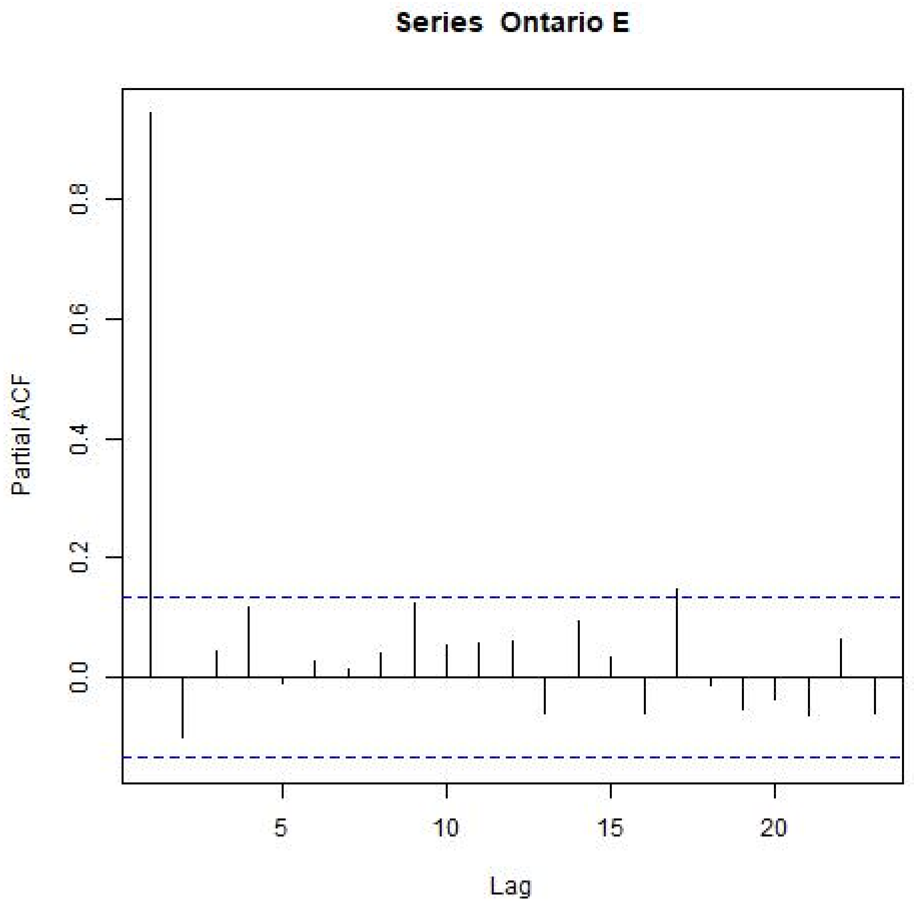
Series Ontario E
Partial ACF
0.0
0.2
0.4
0.6
0.8
5
10
15
20
Lag

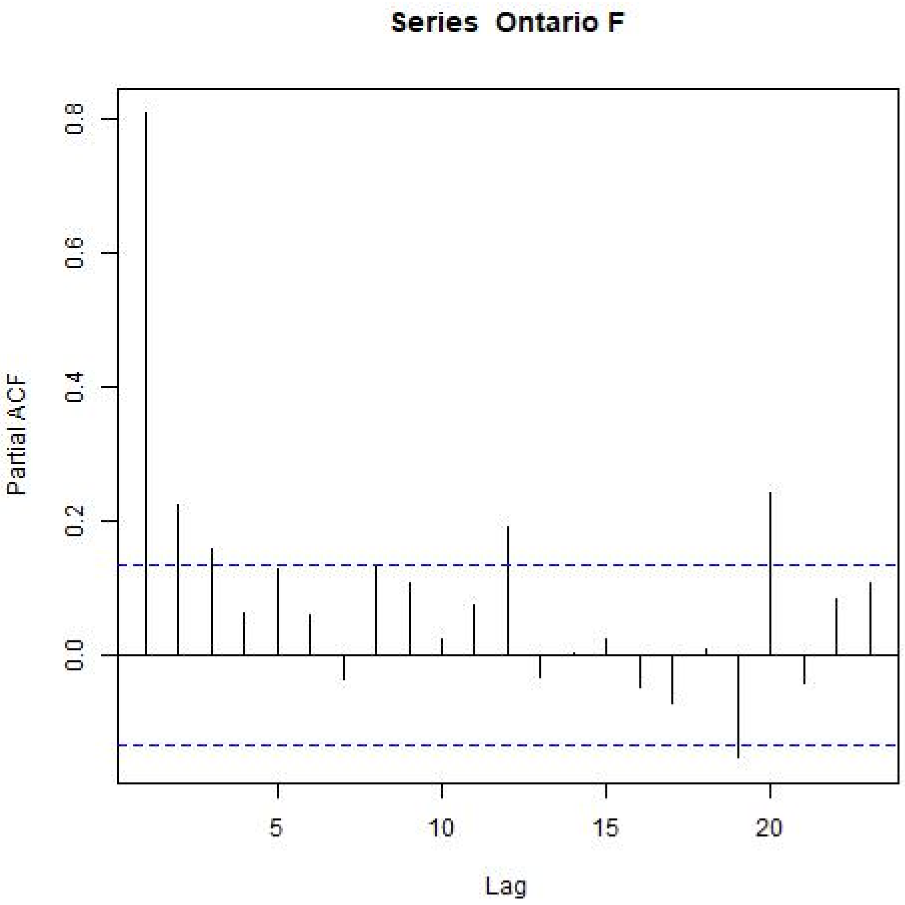

Series Ontario F
Partial ACF
0.0
0.2
0.4
0.6
0.8
5
10
15
20
Lag

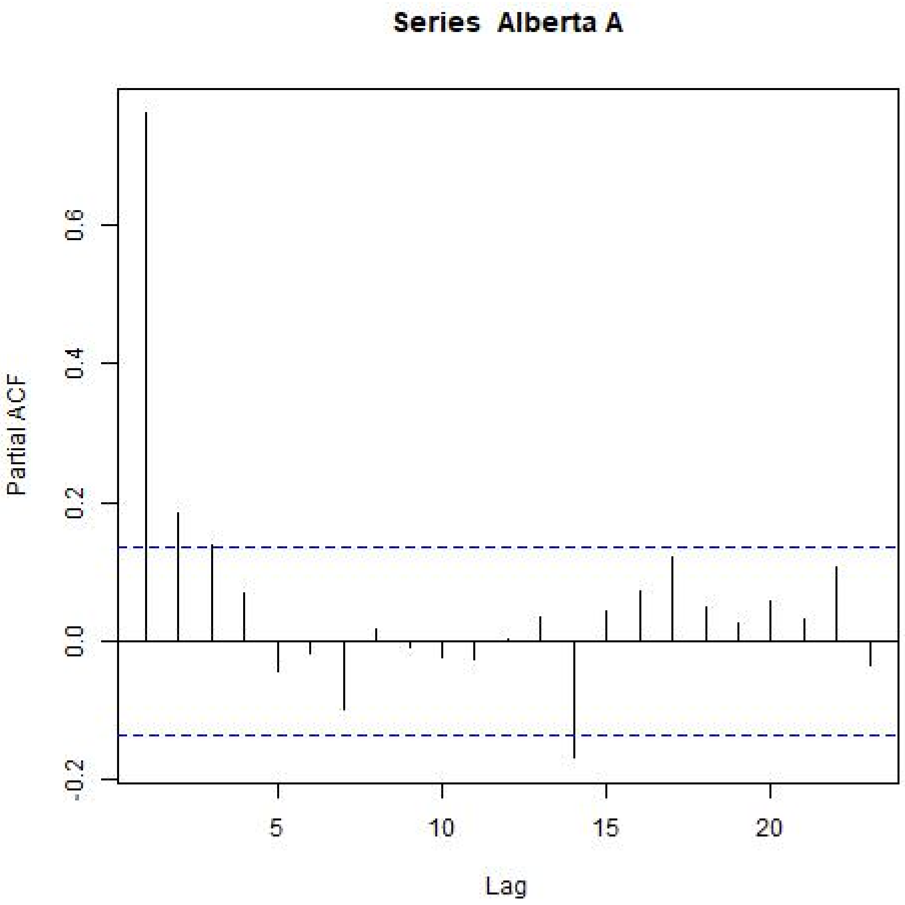
Series Alberta A
Partial ACF
0.6
0.4
0.2
0.0
-0.2
5
10
15
20
Lag

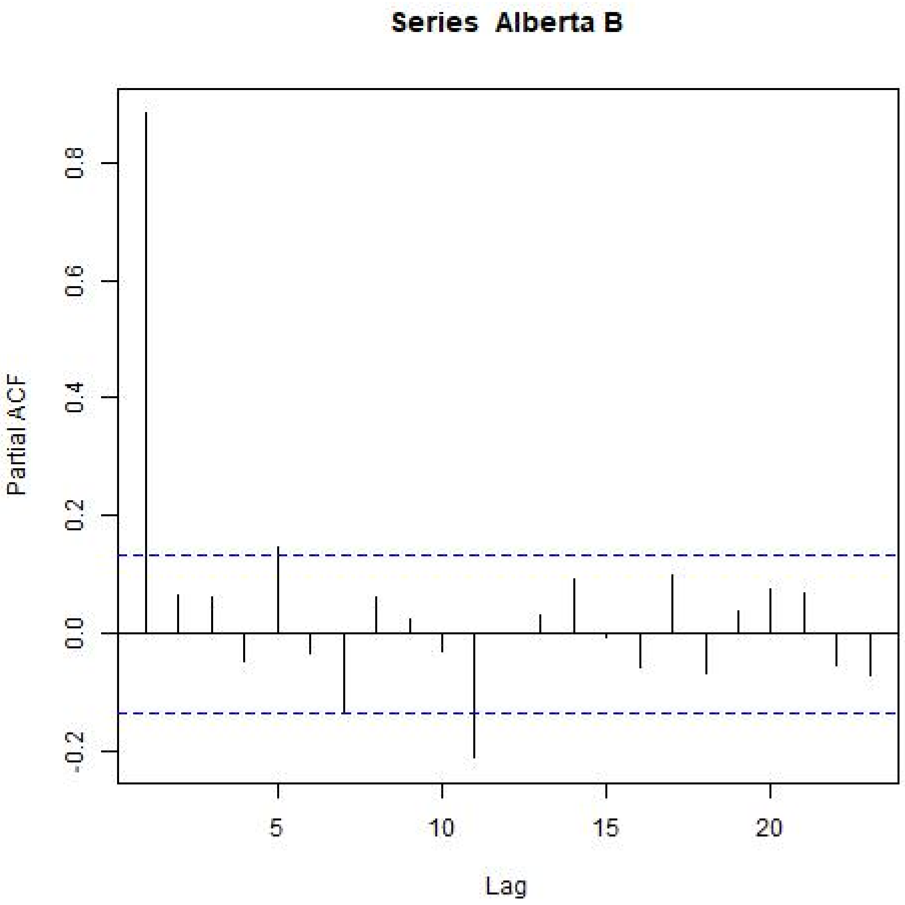

Series Alberta B
Partial ACF
-0.2
0.0
0.2
0.4
0.6
0.8
5
10
15
20
Lag

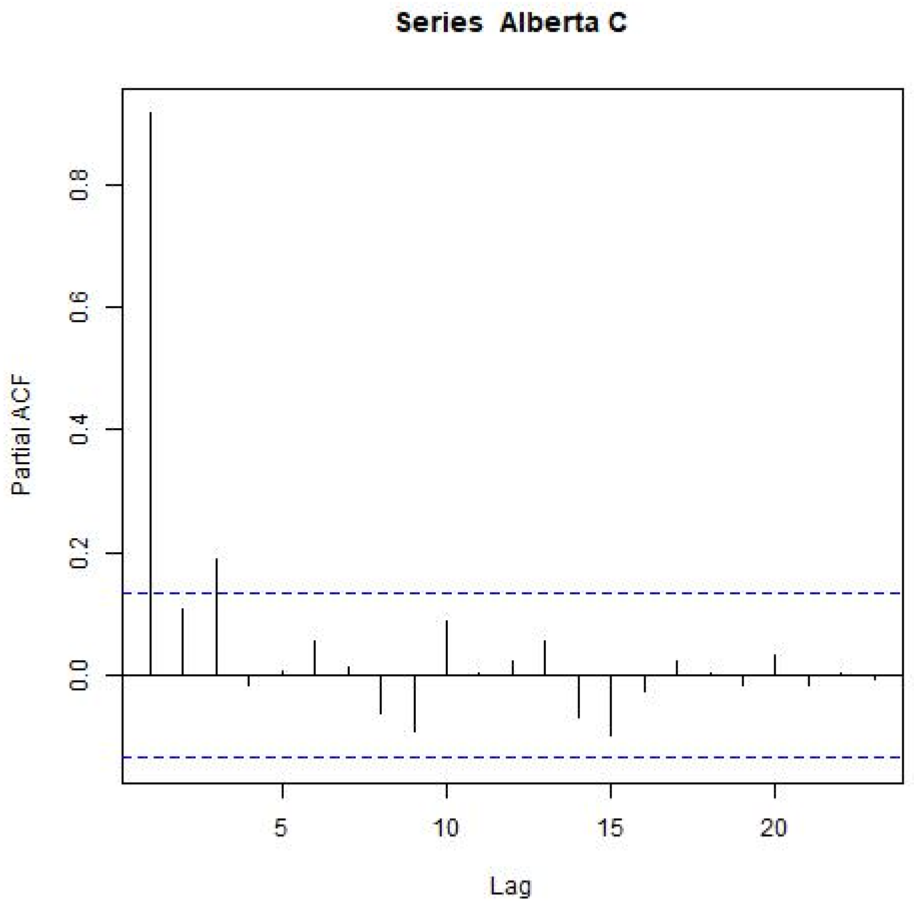

Series Alberta C
Partial ACF
0.0
0.2
0.4
0.6
0.8
5
10
15
20
Lag

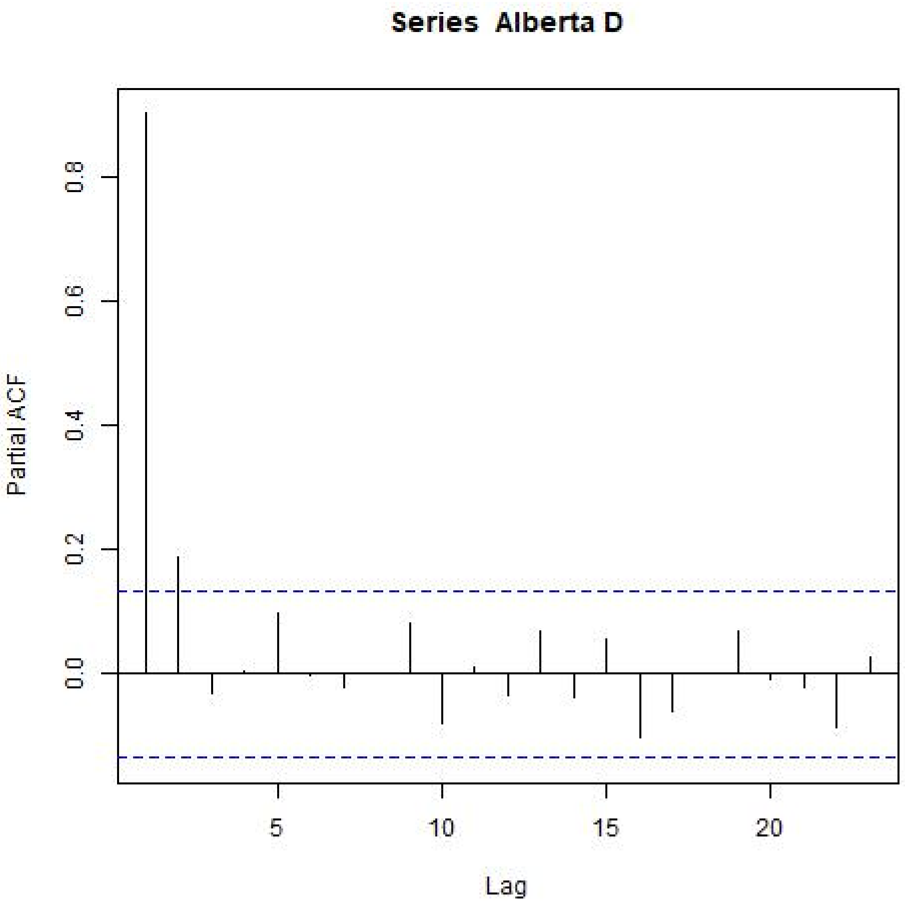

Series Alberta D
Partial ACF
0.0
0.2
0.4
0.6
0.8
5
10
15
20
Lag
