# Supplementary figures and images for: Chemical and physical restraint use during acute care hospitalization of older adults: A retrospective cohort study and time series analysis
Source: PLoS One. 2022 Oct 26;17(10):e0276504. doi: 10.1371/journal.pone.0276504 (PMC9604990; doi:10.1371/journal.pone.0276504)

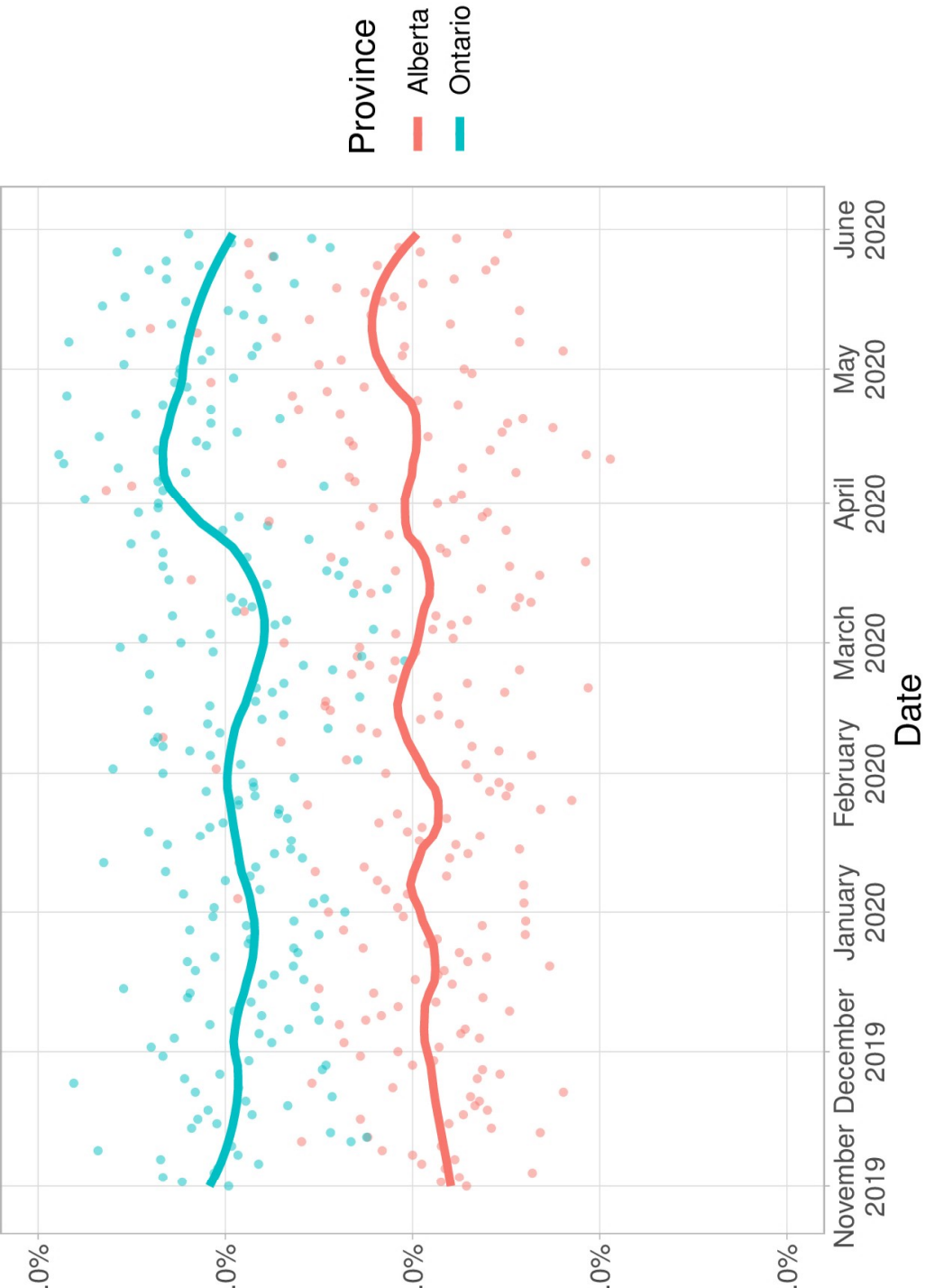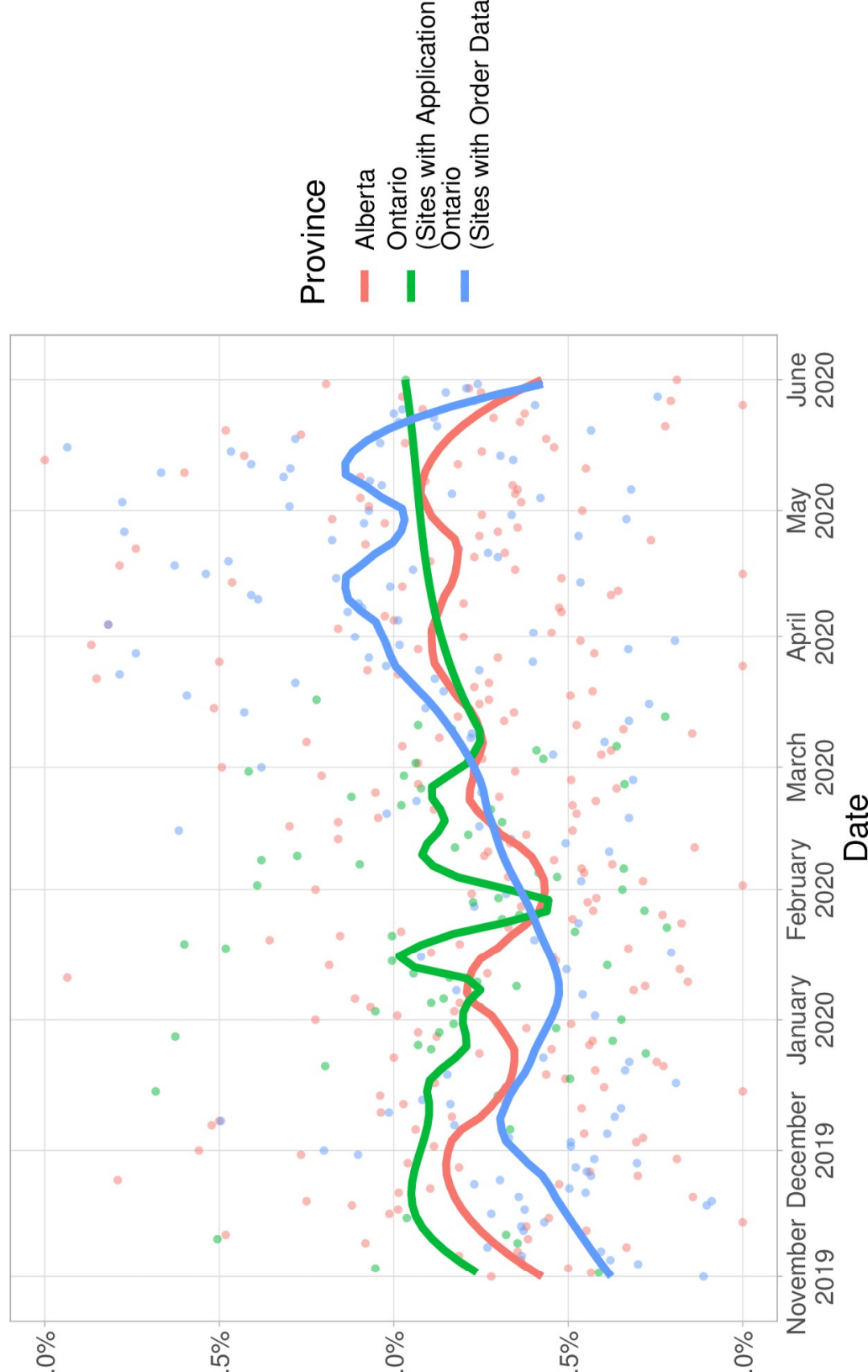

Supplement: S2 Fig — (PDF) [file pone.0276504.s008.pdf]
